# ARPES Signatures of Few-Layer Twistronic Graphenes

**DOI:** 10.1021/acs.nanolett.3c01173

**Published:** 2023-05-26

**Authors:** James E. Nunn, Andrew McEllistrim, Astrid Weston, Aitor Garcia-Ruiz, Matthew D. Watson, Marcin Mucha-Kruczynski, Cephise Cacho, Roman V. Gorbachev, Vladimir I. Fal’ko, Neil R. Wilson

**Affiliations:** † Diamond Light Source, Division of Science, Didcot OX11 0DE, U.K.; ‡ Department of Physics, University of Warwick, Coventry CV4 7AL, U.K.; § School of Physics and Astronomy, University of Manchester, Oxford Road, Manchester M13 9PL, U.K.; ∥ National Graphene Institute, University of Manchester, Booth St East, Manchester M13 9PL, U.K.; ⊥ Centre for Nanoscience and Nanotechnology, Department of Physics, University of Bath, Bath BA2 7AY, U.K.

**Keywords:** graphene, twistronics, moiré, flat band, angle-resolved photoemission spectroscopy

## Abstract

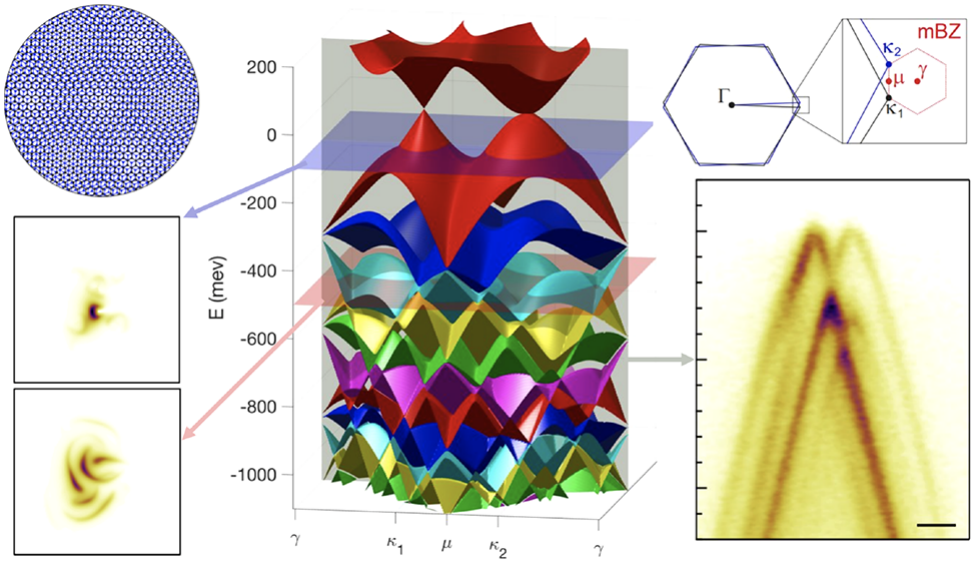

Diverse emergent correlated electron phenomena have been observed in twisted-graphene layers. Many electronic structure predictions have been reported exploring this new field, but with few momentum-resolved electronic structure measurements to test them. We use angle-resolved photoemission spectroscopy to study the twist-dependent (1° < θ < 8°) band structure of twisted-bilayer, monolayer-on-bilayer, and double-bilayer graphene (tDBG). Direct comparison is made between experiment and theory, using a hybrid **k**·**p** model for interlayer coupling. Quantitative agreement is found across twist angles, stacking geometries, and back-gate voltages, validating the models and revealing field-induced gaps in twisted graphenes. However, for tDBG at θ = 1.5 ± 0.2°, close to the magic angle θ = 1.3°, a flat band is found near the Fermi level with measured bandwidth *E*_w_ = 31 ± 5 meV. An analysis of the gap between the flat band and the next valence band shows deviations between experiment (Δ_h_ = 46 ± 5 meV) and theory (Δ_h_ = 5 meV), indicative of lattice relaxation in this regime.

Reports of anomalous superconductivity^1^ and correlated insulating behavior^2^ in magic-angle twisted-bilayer graphene (MATBG) sparked an avalanche of research into magic-angle effects in two-dimensional materials (2DMs) and into 2D twistronics more generally. Overlapping two identical 2D crystal lattices with a small angular rotation (twist angle, θ) between them creates a long-range moiré superlattice. For MATBG, moiré interactions between the layers create a flat band near the Fermi level^3^ whose filling can be electrostatically controlled by gate electrodes. The high density of states within this flat band results in strong, and gate-tunable, electron correlation effects,^1,2,4^ as also observed in twisted-bilayer transition-metal dichalcogenides^5,6^ and in twisted few-layer graphenes.^7−12^ However, there are challenges to modeling these systems. The large number of atoms in a moiré cell complicate the application of *ab initio* approaches, leading to the development of various multiscale approaches^13^ such as large-scale density functional theory,^14−16^ tight-binding and continuum models.^3,17,18^ Although these give qualitatively similar predictions, the details of the dispersions, and hence their properties, depend on the simulation methodology and parameter set used. Experimental studies are therefore vital to validate and refine the theoretical models and to understand the electronic band structure changes which underlie the emergent twistronic effects.

Angle-resolved photoemission spectroscopy (ARPES) gives unique insight into the momentum-resolved electronic band structure of 2DMs and 2D heterostructures.^19−25^ Due to the short mean free path of the photoexcited electrons, ARPES is sensitive to the top few atomic layers, enabling the study of layer-dependent effects, while *in situ* back gating of 2D heterostructures during ARPES allows the study of band structure changes with carrier concentration^26−28^ and with transverse displacement fields.^29,30^ ARPES has previously been applied to the study of twisted graphenes, initially studying multilayer graphene grown on SiC or copper, where twisted regions can be found by chance.^31−35^ However, interactions with the substrate cause complications such as inhomogeneous doping, increased screening, and additional moiré periodicities. Instead, mechanical exfoliation and stacking on boron nitride can be used to fabricate twisted-graphene samples at defined twist angles for ARPES, for example, showing that moiré superlattice effects persist even in large-angle twisted-bilayer graphene where the moiré period is short.^36^ Also using this approach, recent reports of ARPES on MATBG detected a flat band at the Fermi level,^37,38^ although neither the flat band dispersion nor the Fermi surface topology could be resolved.

Here, we use a direct comparison between simulated and measured ARPES spectra to test electronic band structure predictions for few-layer graphene samples with different stacking geometries and over a range of (small) twist angles. Our measurements visualize the twist-dependent electronic band structure, giving a quantitative test of the validity of the hybrid **k**·**p** model and the corresponding choice of empirical parameters. We measure the dispersion of the flat valence band near the Fermi level in twisted-double-bilayer graphene, finding small but significant differences between the experimental results and the hybrid **k**·**p** model simulations. Extending this, using ARPES with *in situ* gating we show that, away from this magic-angle regime, the model accurately describes the gate-dependent behavior: applying a back-gate voltage results in both electrostatic doping of the graphene layers and a displacement field across them, that opens a field-dependent gap at the Dirac point of bilayer graphene.

Twisted-few-layer graphene samples were fabricated on hexagonal boron nitride (hBN) by a modified tear-and-stack approach,^39,40^ as described in the methods summary in section 1 in the Supporting Information (SI) and in detail in section 2 in the SI. A graphite back-gate electrode was incorporated into some of the devices, shown schematically in Figure 1a. Scanning probe microscopy and scanning photoemission microscopy showed homogeneous regions a few micrometers across in the twisted graphenes. Spatially resolved ARPES spectra were acquired from within these regions at the nanoARPES branch of the I05 beamline at Diamond Light Source, as described in section 3 in the SI. Energies are measured relative to the Fermi energy, *E*_F_, determined by fitting the drop in photoemission intensity at *E*_F_ on a metal electrode connected to the graphene stack.

**Figure 1:**
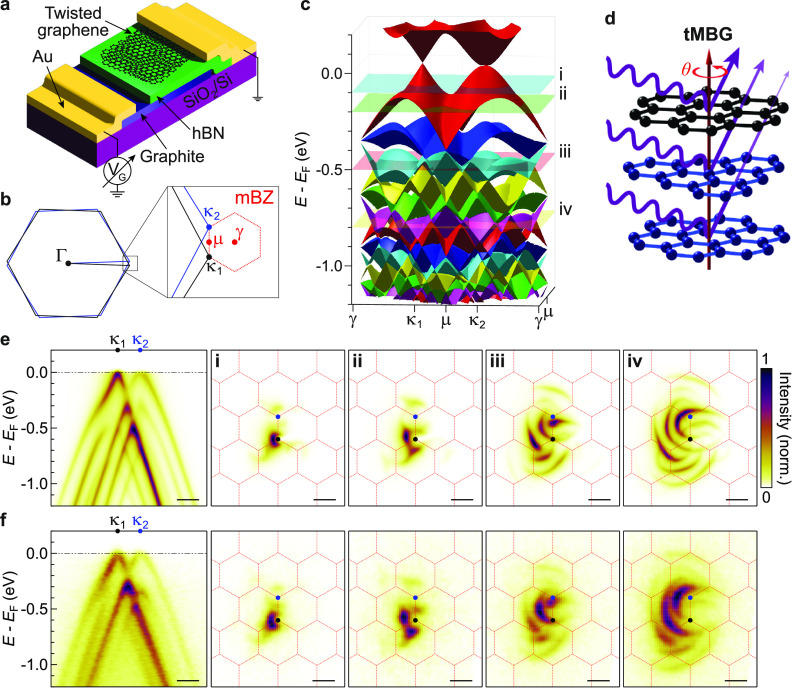
Comparison of simulated and measured ARPES spectra for tMBG. Schematic of (a) the twisted graphene heterostructures and (b) the twisted graphene Brillouin zones and corresponding moiré Brillouin zone (mBZ). (c) Band structure for tMBG at θ = 3.4° computed using the hybrid **k**·**p** model. (d) Schematic of the photoemission process in multilayered graphene. Photoelectrons from deeper layers are attenuated due to scattering in the material. (e) Simulated and (f) experimental ARPES spectra for tMBG at θ = 3.4°. Left-hand panels show energy-momentum cuts taken along the κ_1_ → κ_2_ direction shown in **b**, where κ_1_ corresponds to the upper layer. Panels **i-iv** are constant energy maps at *E* – *E*_F_ = (i) −100 meV, (ii) −200 meV, (iii) −500 meV, and (iv) −800 meV, as marked by the horizontal planes in (c). All scale bars are 0.1 Å^–1^.

A hybrid **k**·**p** theory-tight-binding model was used to calculate the electronic structure of twisted-few-layer graphenes, using a full set of Slonczewski–Weiss–McClure (SWMcC) parameters for the aligned multilayer graphenes.^41−43^ The values of the SWMcC parameters, as given in Table 1 of the SI, were established by earlier transport studies^44^ and are used here without fitting to the experimental data. Details of the calculations are given in section 4 in the SI. We focus on the electronic band structure across the moiré Brillouin zones (mBZs), at the graphene Brillouin zone corners (Figure 1b). Band folding results in a rich electronic band structure, as shown in Figure 1c for twisted-monolayer-on-bilayer graphene (tMBG) at a twist angle of θ = 3.4°.

Not all of these bands are apparent in ARPES spectra, and their relative intensities change with measurement conditions. The photoemission intensity depends on matrix elements for the photoexcitation process, resonance and interference effects, and attenuation of the photoemitted electrons.^45^ For complex systems, this can lead to confusion over the interpretation of the ARPES spectra and their relation to electronic band structure calculations, necessitating simulation of the ARPES intensity. To do this, the probability of a photostimulated transition from an initial band state in graphene calculated using a hybrid **k**·**p** method to a plane wave state in vacuum was calculated using Fermi’s Golden Rule,^34,46^ as described in section 5 in the SI. The final state (ψ_vac_) was assumed to be a plane wave in the vacuum. Travel of the photoelectron to the surface and escape and detection were included by accounting for an increased path length for emission from the lower layers, resulting in a phase difference in the plane waves and an attenuation in intensity, as shown schematically in Figure 1d. This phase difference was determined from the out-of-plane component of the final state momentum, *k*_*z*_; the validity of this approach was tested by comparison of simulation to measurement for photon-energy-dependent spectra of bilayer graphene (see section 5 in the SI). Finally, the simulated spectra were convoluted by a Lorentzian peak of width 60 meV to account for experimental broadening^47^ due to sample quality, intrinsic line width, and measurement resolution.

Simulated ARPES spectra for tMBG at θ = 3.4° are given in Figure 1e and compared to the corresponding experimental spectra in Figure 1f, for which the measured twist angle is θ = 3.4 ± 0.1°. The twist angle was determined from constant energy maps near *E*_F_, using the replica bands to determine the mBZ and hence θ, as described in section 6 in the SI. Energy-momentum slices through the corners of the mBZ show the Dirac cones of the primary bands, moiré replica bands, and hybridization gaps where bands from the rotated monolayer anticross with those of the bilayer. Photoemission from bands in the upper monolayer graphene (MLG) is more intense than from those in the bilayer graphene (BLG) underneath, with the replica bands being lower in intensity than the corresponding primary bands.

In the ARPES constant energy maps, plotted over the mBZs (shown in red) in Figure 1e, the primary and replica bands are readily identified near *E*_F_, with the Dirac cones at the mBZ κ points. However, at deeper energy cuts, interactions between bands make it harder to assign the origin of the photoemission intensity to a specific band in a given layer. The band decomposition at these constant energy slices, determined from the electronic band structure calculations, is shown for comparison in section 7 in the SI. Despite this complexity, it is clear that the model accurately captures both the relative spectral intensities and band positions of the experimental spectra, enabling the electronic structure to be probed in greater detail.

Comparison of spectra acquired from different twist angles and stacking orders allows a quantitative test of band structure predictions from the ARPES data. In Figure 2, ARPES energy-momentum slices are presented for twisted-bilayer graphene (tBG), twisted-monolayer-on-bilayer graphene and twisted-double-bilayer graphene (tDBG). The twist angle is defined relative to Bernal stacking.

**Figure 2:**
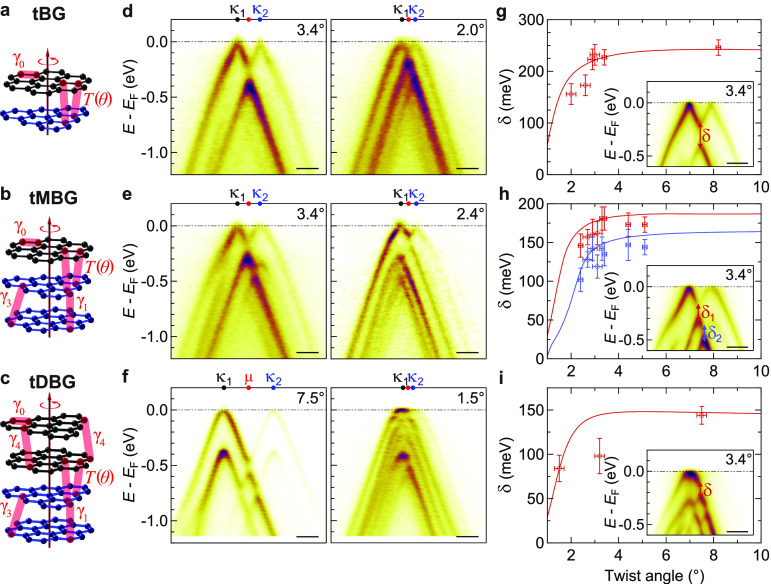
Twist angle and layer number dependence of ARPES energy momentum spectra. (a–c) Schematics of tBG, tMBG, and tDBG, respectively, labeled with the inter- and intralayer coupling parameters. (d–f) Experimental ARPES energy-momentum cuts in the κ_1_–κ_2_ direction for tBG, tMBG, and tDBG, respectively, at 2 twist angles. (g–i) Plots of hybridization gap size vs twist angle for tBG, tMBG, and tDBG, respectively. The solid lines correspond to the data extracted from the simulated spectra, and the data points to those from the experimental spectra. Insets show simulated ARPES spectra for the corresponding layer geometries, at θ = 3.4°, with the hybridization gaps labeled. All scale bars are 0.1 Å^–1^. The first panel in (e) is from monolayer-on-bilayer graphene, and the second is from bilayer-on-monolayer graphene.

For larger twist angles, θ > 3°, the primary bands are readily resolved in the ARPES spectra with only faint replica bands, while at smaller twist angles the intensities of the replica bands increase and the electronic band structure becomes more complex. Quantitative analysis of the replica band intensities near *E*_F_ is given in section 8 in the SI for tBG at different twist angles, showing good agreement between the experimental data and the simulations. In general, the intensity of the replicas decreases in successive mBZs away from the primary bands, as expected due to the lower probability of scattering further in reciprocal space.

Where the bands meet, hybridization between them results in anticrossings, with the gaps most obvious at or near the μ point in the slices shown here. The size of the gaps (δ) at the anticrossings of the primary bands are plotted in Figure 2g–i as a function of the twist angle. These were determined by fitting of energy distribution curves (EDCs), as illustrated in the insets of Figure 2 and described in detail in section 9 in the SI. δ depends on both the interlayer and intralayer coupling parameters; hence, the agreement between experiment (data points) and theory (solid lines) across all twist angles and stacking arrangements demonstrates the accuracy of the theoretical approach for describing the twisted interface. Note that the same SWMcC parameters were used for each structure, without fitting to the experimental data.

At larger twist angles, θ ≥ 4°, the simulations predict that δ is roughly constant with twist angle, verified by the agreement to the experimental results. In this regime, the anticrossings occur at energies at which the bands are well described by a linear dispersion and hence δ scales only with the strength of the potential that couples the states in the different layers. In these simulations, the magnitude of variation of the moiré potential is a constant factor independent of twist angle, related to the interlayer coupling parameters. However, at smaller θ, the magnitude of the hybridization gap depends sensitively on twist angle and changes subtly with stacking geometry (Figures 2g–i). At these small twist angles, the anticrossings lie close to the Dirac points and distort the linear dispersion, as can be seen in the ARPES spectra, forming a band whose bandwidth decreases with twist angle.

In the smallest twist angle sample measured here, tDBG at θ = 1.5 ± 0.2°, an almost flat valence band is observed at *E*_F_ (Figure 2f, right-hand panel). A more detailed analysis of this band is shown in Figure 3. Energy-momentum cuts through the high-symmetry points (Figure 3a–c with their directions indicated on the constant energy plot in Figures 3d) show ARPES intensity near *E*_F_ in all directions, corresponding to the flat band, with a clear gap to the lower lying valence band states.

**Figure 3:**
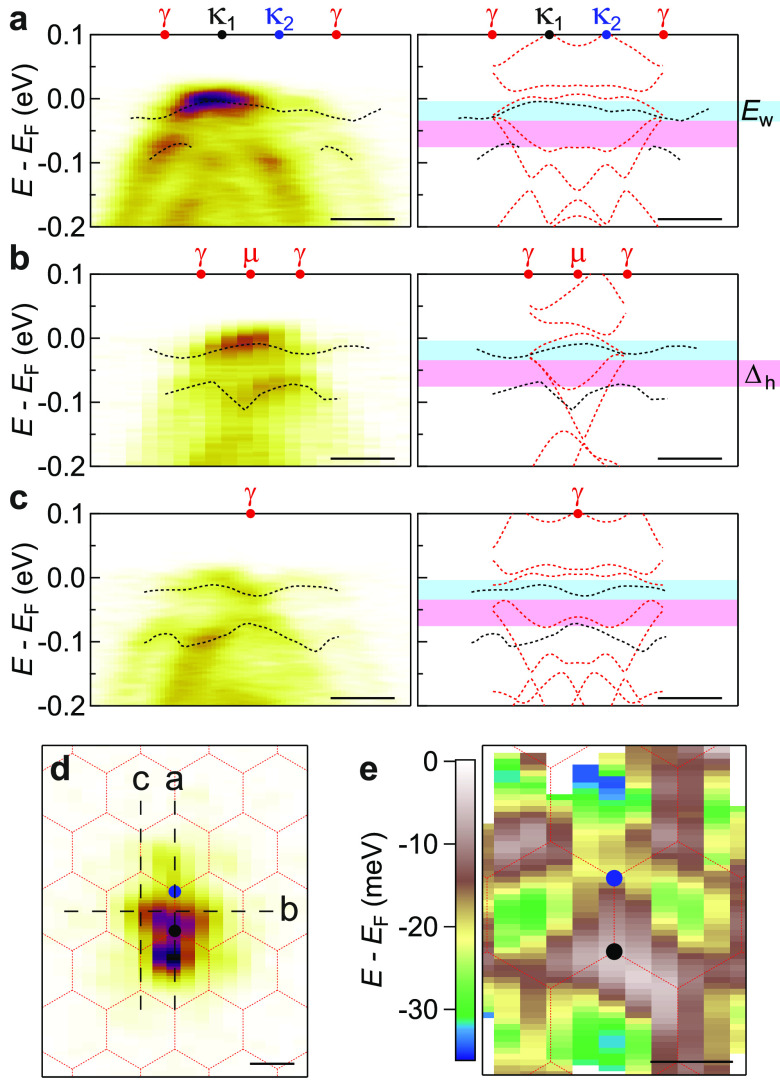
Flat band dispersion in 1.5° tDBG. (a–c) Energy-momentum cuts (left) along the black dashed lines in (d) and the corresponding band dispersions (right). The black lines correspond to the peak positions extracted from the experimental data by fitting EDCs and the red lines corresponding to the predicted electronic structure. (d) ARPES constant energy plot at *E* – *E*_F_ = −30 meV with the mBZs overlaid in red. (e) Energy of the flat band plotted in the *k*_*x*_–*k*_*y*_ plane, with the mBZs overlaid in red. All scale bars are 0.05 Å^–1^.

The band dispersion across the first few mBZs was found from the experimental spectra by fitting EDCs and is shown by the black lines in the right-hand panels of Figure 3a-c, with the predicted band structure in red. Figure 3e shows the energy of this valence band edge as a *k*_*x*_–*k*_*y*_ plot; the corresponding plot from the simulations is shown in section 10 in the SI. Although it is at the limit of the experimental resolution here, the flat band has a weak dispersion, periodic across the mBZ as required, with the band minimum at γ, and is clearly gapped from the lower lying valence bands across all of the mBZ.

The key band parameters can be determined from these data. The bandwidth of the flat valence band at *E*_F_ is measured to be *E*_w_ = 31 ± 5 meV, in good agreement with the predicted value from the electronic structure calculations of *E*_w_ = 33 meV. The band gap to the next occupied valence band state is smallest at γ, where it is measured to be Δ_h_ = 46 ± 5 meV, significantly greater than the predicted value of Δ_h_ = 5 meV. Note that the electronic structure calculations here do not incorporate the effects of lattice relaxation, which are expected to be significant in determining the low-energy electronic structure in twisted graphenes for small θ, close to or below the magic angle.^13,46^ For tDBG at θ = 1.5°, Haddadi et al. found that the gap at γ increased by roughly an order of magnitude from Δ_h_ ≈ 5 meV to Δ_h_ ≈ 40 meV when lattice relaxation was included,^48^ consistent with our experimentally determined value. The bandwidth is predicted to decrease further to *E*_w_ ≈ 5 meV at the magic angle of θ = 1.3°, with the gap staying roughly similar in magnitude. A spectrally isolated flat band such as this has been proposed to be favorable for the emergence of correlated insulators,^49^ and these results prove that, despite previous reports,^50,51^ a vertical displacement field is not required to produce such a band in tDBG.

Integrating a back-gate electrode into the tMBG heterostructure, as shown schematically in Figure 1a, allows the gate dependence of the electronic band structure to be investigated. ARPES spectra at varying *V*_G_ for a 3.4 ± 0.1° tMBG sample (MLG on top, BLG closer to the gate electrode), with an hBN dielectric thickness of *d* = 26 nm, are shown in Figure 4a. For a positive applied back-gate voltage, *V*_G_ > 0, the Dirac point energies move below *E*_F_, corresponding to *n*-doping. There is no apparent broadening of the spectra, indicating a uniform applied field. Consistent with it being the lower layer, closer to the gate electrode, the Dirac point of the BLG, at momentum κ_2_ and energy *E*_D_^BL^, shifts more than that of the MLG, at momentum κ_1_ and energy *E*_D_^ML^. This indicates partial screening of the back gate^52^ and a displacement field across the twisted graphene layers which also opens an energy gap, Δ, at the Dirac point of the BLG.^53,54^ The shift of the BLG bands relative to those of the MLG means that the band anticrossings occur at slightly different energies and momenta, subtly changing the interactions between bands and hybridization between layers.

**Figure 4:**
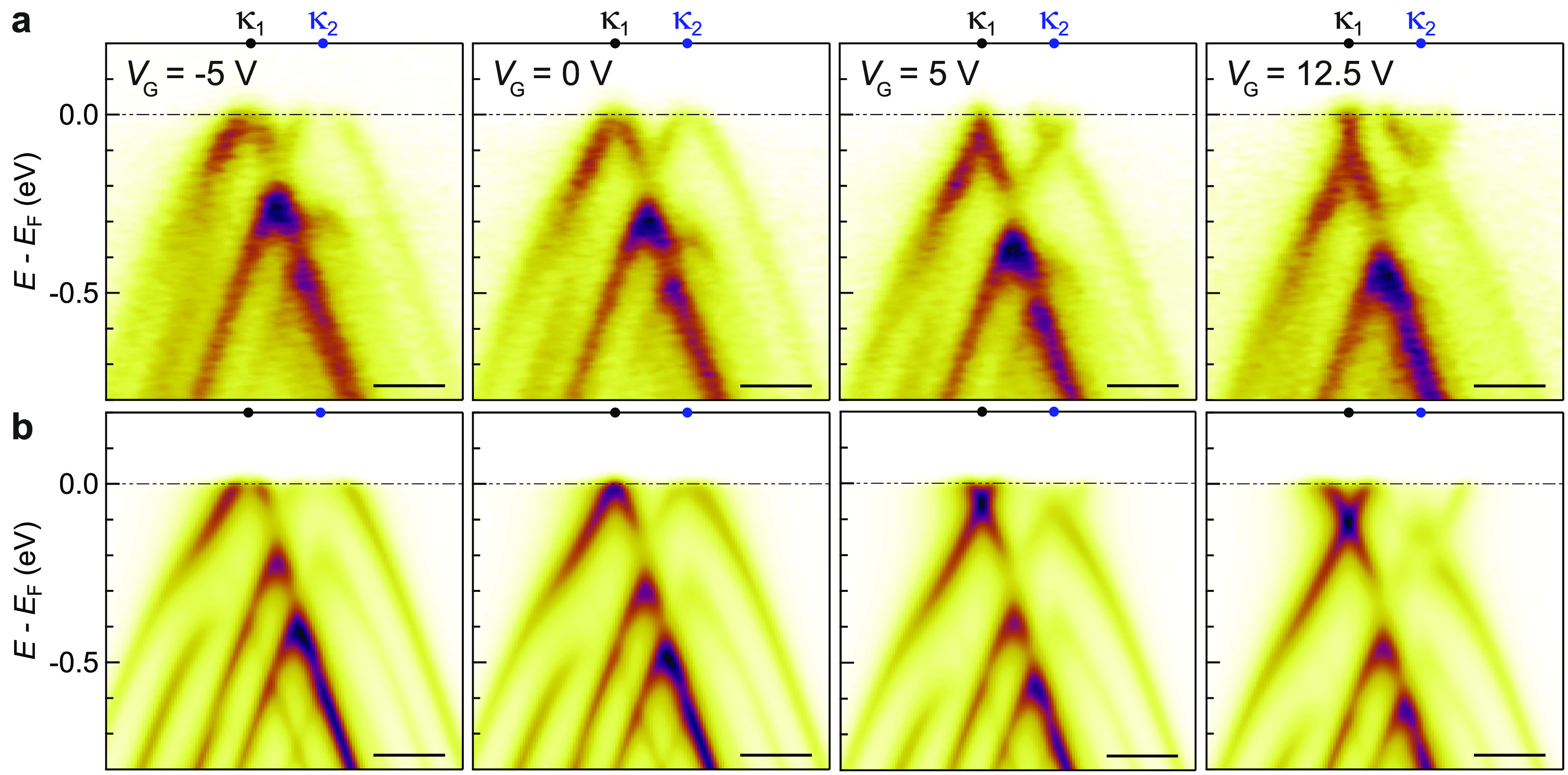
Electrostatic gating of tMBG. (a) ARPES energy-momentum cuts of 3.4 ± 0.1° tMBG taken along the κ_1_–κ_2_ direction taken at the labeled gate voltages. (b) Simulated ARPES spectra for 3.4° tMBG at varying back-gate voltages, as labeled. All scale bars are 0.1 Å^–1^.

We incorporate the effect of electrostatic gating into the simulations using a self-consistent analysis of on-layer potentials.^52^ Starting from the band structure without applied field, potential differences between the layers are added that are proportional to the applied field. The charge redistribution across the layers is determined, and the charge density in each layer is used to calculate the screening fields and the resultant modified interlayer potentials. The band structure is recalculated using these modified interlayer potentials and the process iterated to convergence to give a self-consistent response to the applied *V*_G_ (see section 11 in the SI for further details). Simulated ARPES spectra at varying *V*_G_ are shown in Figure 4b, for the same sample geometry as the experimental data (tMBG at θ = 3.4° with a hBN dielectric thickness of *d* = 26 nm, and hBN dielectric constant ε = 4^55^).

Changes to the band dispersion with *V*_G_ are shown in Figure 5a, emphasizing the dominant effects of the applied field: *E*_D_^BL^ and *E*_D_^ML^ shift relative to *E*_F_, indicating electrostatic doping in both layers; *E*_D_^BL^ shifts relative to *E*_D_^ML^, consistent with a field transverse to the layers; this field opens a gap, Δ, at the Dirac point of the bilayer graphene.^53,54^ These key parameters were determined by fitting of the spectra. The change in Dirac point energies is plotted in Figure 5b (solid line from the simulations, data points from the experiment, blue corresponds to BLG and black to MLG). For *V*_G_ > 0, *E*_D_^BL^ – *E*_F_ < *E*_D_^ML^ – *E*_F_ < 0, corresponding to electron doping, while for *V*_G_ < 0, *E*_D_^BL^ – *E*_F_ > *E*_D_^ML^ – *E*_F_ > 0 corresponding to hole doping. The resultant charge density, *n*, is plotted in Figure 5c. For the simulations, *n* is calculated directly from the electronic band structure models by counting the charge in each layer; the experimental data are calculated from *E*_D_^BL^ and *E*_D_^ML^ as described in section 12 in the SI.

**Figure 5:**
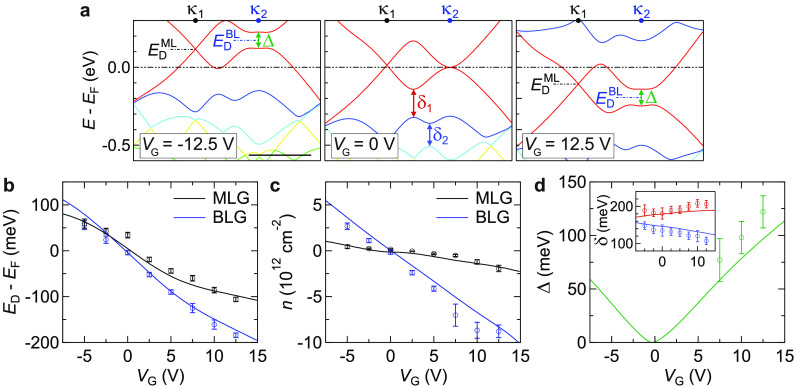
Analysis of band structure changes and doping with *V*_G_ for 3.4° tMBG, on 26 nm hBN. (a) Band structure around the Fermi level for tMBG at different *V*_G_. Labels show the Dirac points (*E*_D_), bilayer gap size (Δ), and hybridization gap sizes (δ). The scale bar is 0.1 Å^–1^. (b, c) Dirac point energies, *E*_D_, and carrier densities, *n*, respectively, as a function of *V*_G_ for the monolayer (black) and bilayer (blue) Dirac cones. (d) The energy gap, Δ, at the bilayer Dirac point as a function of *V*_G_. The inset shows the hybridization gaps, δ_1_ in red and δ_2_ in blue, as a function of *V*_G_. Data points are experimental values extracted from the ARPES data, while solid lines are extracted from the simulations.

The charge densities in the bilayer, *n*_BLG_, and the monolayer, *n*_MLG_, both scale roughly linearly with *V*_G_, but at a lower rate in the monolayer, *n*_MLG_ ≈ *n*_BLG_/4, such that most of the charge is localized in the BLG, screening the MLG from the gate. At *V*_G_ = 15 V there is almost a 100 meV difference between *E*_D_^ML^ and *E*_D_^BL^, corresponding to a strong Stark shift due to the displacement field. This changes the BLG dispersion, opening a band gap that scales roughly linearly with the magnitude of *V*_G_ and at *V*_G_ = 15 V is again on the order of 100 meV. Finally, we note that the shift of the BLG bands relative to those of the MLG results in subtle changes to the interlayer coupling, as can be seen through analysis of the hybridization gaps. For example, the gaps at the anticrossings of the primary bands, δ_1_ and δ_2_ as labeled on the band dispersion in Figure 5a central panel, change in opposite direction with *V*_G_, as shown in the inset of Figure 5d. For all band parameters, there is good agreement between the experimental measurements and the simulations, confirming the validity of the model used.

We note that our results illustrate a challenge to applying ARPES with in situ gating to the study of, for example, filling-factor-dependent band renormalization in MATBG: a back gate does not just tune the Fermi level, it also applies a transverse field that subtly changes the hybridization between layers and hence the Fermi surface. For transport measurements, top and back gates are simultaneously applied to allow separate control of field and doping. But, due to its surface sensitivity, conventional ARPES cannot interrogate through a top gate. Despite this, ARPES offers a unique capability for resolving the layer-dependent electronic band structure in 2D heterostructures and the evolution of this structure with applied electric field, a crucial control parameter for 2D devices. With further advances in sample fabrication and ARPES resolution, we expect that investigation of filling-factor-dependent correlated electron phases in twisted graphenes will be imminently achievable.

Through comparison between measured and simulated ARPES spectra, we have tested the validity of the hybrid **k**·**p** theory tight-binding model for predicting the electronic band structure of twisted-few-layer graphenes in the small twist-angle regime. The simulated spectra quantitatively agree with the measurements, from not only their band dispersions but also their spectral weights, across a range of twist angles (1° < θ < 8°) and numbers of layers (tBG, tMBG, tDBG) with a single set of empirically derived parameters describing the inter- and intralayer coupling for twisted and aligned layers. A detailed analysis of the flat band dispersion in twisted-double-bilayer graphene at θ = 1.5 ± 0.2°, close to the magic angle of 1.3°,^48^ shows that although there is close agreement between the hybrid **k**·**p** model and the experimental data for the width of the valence band at the Fermi energy, at *E*_w_ ≈ 30 meV, the gap to the lower lying valence band states is larger than predicted, Δ_h_ = 46 ± 5 meV, consistent with the importance of lattice relaxation effects at twist angles close to the magic angle. ARPES with in situ gating reveals the evolution of the electronic band structure with the application of a back-gate electrode, demonstrating the importance of both doping and transverse electric field, with quantitative agreement to predicted spectra achieved through a self-consistent approach to modeling the electronic band structure changes with gating. The results reinforce the importance of Stark shifts in 2D heterostructures, even for metallic 2D materials. With this validation, the models can be used with confidence to explore the electronic band structure and emergent transport and optical properties of twisted-few-layer graphenes.

## Data Availability

The data that support the plots in the manuscript are available from the corresponding authors upon reasonable request.

## Supporting Information Available

Description of methods for sample fabrication, ARPES, continuum model, simulating ARPES intensity, determining the twist angle, analysis of spectral features associated with band number, of replica band intensity, of hybridization gaps in EDCs, self-consistent analysis of the effect of a back-gate voltage, and of gated tMBG Dirac cones (PDF)

## References

[ref1] Cao Y.; et al. Unconventional superconductivity in magic-angle graphene superlattices. Nature 2018, 556, 43–50. 10.1038/nature26160. 29512651

[ref2] Cao Y.; et al. Correlated insulator behaviour at half-filling in magic-angle graphene superlattices. Nature 2018, 556, 80–84. 10.1038/nature26154. 29512654

[ref3] Bistritzer R.; MacDonald A. H. Moire bands in twisted double-layer graphene. Proc. Natl. Acad. Sci. U.S.A. 2011, 108, 12233–12237. 10.1073/pnas.1108174108. 21730173 PMC3145708

[ref4] Choi Y.; et al. Electronic correlations in twisted bilayer graphene near the magic angle. Nat. Phys. 2019, 15, 1174–1180. 10.1038/s41567-019-0606-5.

[ref5] Wang L.; Shih E.-M.; Ghiotto A.; Xian L.; Rhodes D. A.; Tan C.; Claassen M.; Kennes D. M.; Bai Y.; Kim B.; Watanabe K.; Taniguchi T.; Zhu X.; Hone J.; Rubio A.; Pasupathy A. N.; Dean C. R.; et al. Correlated electronic phases in twisted bilayer transition metal dichalcogenides. Nat. Mater. 2020, 19, 861–866. 10.1038/s41563-020-0708-6. 32572205

[ref6] Zhang Z.; et al. Flat bands in twisted bilayer transition metal dichalcogenides. Nat. Phys. 2020, 16, 1093–1096. 10.1038/s41567-020-0958-x.

[ref7] Chen S.; et al. Electrically tunable correlated and topological states in twisted monolayer-bilayer graphene. Nat. Phys. 2021, 17, 374–380. 10.1038/s41567-020-01062-6.

[ref8] Xu S.; et al. Tunable van Hove singularities and correlated states in twisted monolayer–bilayer graphene. Nat. Phys. 2021, 17, 619–626. 10.1038/s41567-021-01172-9.

[ref9] Cao Y.; et al. Tunable correlated states and spin-polarized phases in twisted bilayer-bilayer graphene. Nature 2020, 583, 215–220. 10.1038/s41586-020-2260-6. 32499644

[ref10] Shen C.; et al. Correlated states in twisted double bilayer graphene. Nat. Phys. 2020, 16, 520–525. 10.1038/s41567-020-0825-9.

[ref11] Zhang C.; et al. Visualizing delocalized correlated electronic states in twisted double bilayer graphene. Nat. Commun. 2021, 12, 2516 10.1038/s41467-021-22711-1. 33947845 PMC8096954

[ref12] Liu X.; et al. Spectroscopy of a tunable moiré system with a correlated and topological flat band. Nat. Commun. 2021, 12, 2732 10.1038/s41467-021-23031-0. 33980832 PMC8115081

[ref13] Carr S.; Fang S.; Kaxiras E. Electronic-structure methods for twisted moiré layers. Nat. Rev. Mater. 2020, 5, 748–763. 10.1038/s41578-020-0214-0.

[ref14] Trambly de Laissardiere G.; Mayou D.; Magaud L. Localization of Dirac Electrons in Rotated Graphene Bilayers. Nano Lett. 2010, 10, 804–808. 10.1021/nl902948m. 20121163

[ref15] Trambly de Laissardière G.; Mayou D.; Magaud L. Numerical studies of confined states in rotated bilayers of graphene. Phys. Rev. B 2012, 86 (12), 125413 10.1103/PhysRevB.86.125413.

[ref16] Lucignano P.; Alfè D.; Cataudella V.; Ninno D.; Cantele G. Crucial role of atomic corrugation on the flat bands and energy gaps of twisted bilayer graphene at the magic angle θ∼1.08°. Phys. Rev. B 2019, 99, 195419 10.1103/PhysRevB.99.195419.

[ref17] Lopes dos Santos J. M. B.; Peres N. M. R.; Castro Neto A. H. Graphene Bilayer with a Twist: Electronic Structure. Phys. Rev. Lett. 2007, 99 (25), 256802 10.1103/PhysRevLett.99.256802. 18233543

[ref18] Garcia-Ruiz A.; Deng H.-Y.; Enaldiev V. V.; Fal’ko V. I. Full Slonczewski-Weiss-McClure parametrization of few-layer twistronic graphene. Phys. Rev. B 2021, 104, 085402 10.1103/PhysRevB.104.085402.

[ref19] Bostwick A.; Ohta T.; Seyller T.; Horn K.; Rotenberg E. Quasiparticle dynamics in graphene. Nat. Phys. 2007, 3, 36–40. 10.1038/nphys477.

[ref20] Riley J. M.; et al. Negative electronic compressibility and tunable spin splitting in WSe2. Nat. Nanotechnol. 2015, 10, 1043–1047. 10.1038/nnano.2015.217. 26389661

[ref21] Kim J.; et al. Observation of tunable band gap and anisotropic Dirac semimetal state in black phosphorus. Science 2015, 349, 723–726. 10.1126/science.aaa6486. 26273052

[ref22] Tang S.; et al. Quantum spin Hall state in monolayer 1T’-WTe2. Nat. Phys. 2017, 13, 683–687. 10.1038/nphys4174.

[ref23] Wilson N. R.; et al. Determination of band offsets, hybridization, and exciton binding in 2D semiconductor heterostructures. Sci. Adv. 2017, 3, e1601832 10.1126/sciadv.1601832. 28246636 PMC5298850

[ref24] Katoch J.; et al. Giant spin-splitting and gap renormalization driven by trions in single-layer WS2/h-BN heterostructures. Nat. Phys. 2018, 14, 355–359. 10.1038/s41567-017-0033-4.

[ref25] Jin W.; et al. Tuning the electronic structure of monolayer graphene/ Mo S2 van der Waals heterostructures via interlayer twist. Phys. Rev. B 2015, 92, 201409 10.1103/PhysRevB.92.201409.

[ref26] Nguyen P. V.; et al. Visualizing electrostatic gating effects in two-dimensional heterostructures. Nature 2019, 572, 220–223. 10.1038/s41586-019-1402-1. 31316202

[ref27] Joucken F.; et al. Visualizing the Effect of an Electrostatic Gate with Angle-Resolved Photoemission Spectroscopy. Nano Lett. 2019, 19, 2682–2687. 10.1021/acs.nanolett.9b00649. 30888827

[ref28] Muzzio R.; et al. Momentum-resolved view of highly tunable many-body effects in a graphene/hBN fieldeffect device. Phys. Rev. B 2020, 101, 201409 10.1103/PhysRevB.101.201409.

[ref29] Jones A. J.; et al. Observation of electrically tunable van Hove singularities in twisted bilayer graphene from NanoARPES. Adv. Mater. 2020, 32, 2001656 10.1002/adma.202001656. 32529706

[ref30] Nguyen P. V.; et al. Field-Dependent Band Structure Measurements in Two-Dimensional Heterostructures. Nano Lett. 2021, 21, 10532–10537. 10.1021/acs.nanolett.1c04172. 34851122

[ref31] Kandyba V.; Yablonskikh M.; Barinov A. Spectroscopic characterization of charge carrier anisotropic motion in twisted few-layer graphene. Sci. Rep. 2015, 5, 16388 10.1038/srep16388. 26548567 PMC4637862

[ref32] Razado-Colambo I.; et al. NanoARPES of twisted bilayer graphene on SiC: absence of velocity renormalization for small angles. Sci. Rep. 2016, 6, 27261 10.1038/srep27261. 27264791 PMC4893698

[ref33] Peng H.; et al. Substrate doping effect and unusually large angle van Hove singularity evolution in twisted bi-and multilayer graphene. Adv. Mater. 2017, 29, 1606741 10.1002/adma.201606741. 28481053

[ref34] Thompson J. J. P.; et al. Determination of interatomic coupling between two-dimensional crystals using angle-resolved photoemission spectroscopy. Nat. Commun. 2020, 11, 3582 10.1038/s41467-020-17412-0. 32681042 PMC7367817

[ref35] Iimori T.; et al. Electronic structure of 3°-twisted bilayer graphene on 4H-SiC (0001). Phys. Rev. Mater. 2021, 5, L051001 10.1103/PhysRevMaterials.5.L051001.

[ref36] Hamer M. J.; et al. Moiré superlattice effects and band structure evolution in near-30-degree twisted bilayer graphene. ACS Nano 2022, 16, 1954–1962. 10.1021/acsnano.1c06439. 35073479 PMC9007532

[ref37] Utama M.; et al. Visualization of the flat electronic band in twisted bilayer graphene near the magic angle twist. Nat. Phys. 2021, 17, 184–188. 10.1038/s41567-020-0974-x.

[ref38] Lisi S.; et al. Observation of flat bands in twisted bilayer graphene. Nat. Phys. 2021, 17, 189–193. 10.1038/s41567-020-01041-x.

[ref39] Frisenda R.; et al. Recent progress in the assembly of nanodevices and van der Waals heterostructures by deterministic placement of 2D materials. Chem. Soc. Rev. 2018, 47, 53–68. 10.1039/C7CS00556C. 29111548

[ref40] Kim K.; et al. Van der Waals heterostructures with high accuracy rotational alignment. Nano Lett. 2016, 16, 1989–1995. 10.1021/acs.nanolett.5b05263. 26859527

[ref41] Slonczewski J. C.; Weiss P. R. Band Structure of Graphite. Phys. Rev. 1958, 109, 272–279. 10.1103/PhysRev.109.272.

[ref42] McClure J. W. Band Structure of Graphite and de Haas-van Alphen Effect. Phys. Rev. 1957, 108, 612–618. 10.1103/PhysRev.108.612.

[ref43] McClure J. W. Theory of Diamagnetism of Graphite. Phys. Rev. 1960, 119, 606–613. 10.1103/PhysRev.119.606.

[ref44] Yin J.; et al. Dimensional reduction, quantum Hall effect and layer parity in graphite films. Nat. Phys. 2019, 15, 437–442. 10.1038/s41567-019-0427-6.

[ref45] Damascelli A. Probing the Electronic Structure of Complex Systems by ARPES. Phys. Scr. 2004, T109, 61–74. 10.1238/Physica.Topical.109a00061.

[ref46] Zhu J.; Shi J.; MacDonald A. H. Theory of angle-resolved photoemission spectroscopy in graphene-based moiré superlattices. Phys. Rev. B 2021, 103, 235146 10.1103/PhysRevB.103.235146.

[ref47] Mucha-Kruczyński M.; et al. Characterization of graphene through anisotropy of constant-energy maps in angle-resolved photoemission. Phys. Rev. B 2008, 77, 195403 10.1103/PhysRevB.77.195403.

[ref48] Haddadi F.; Wu Q.; Kruchkov A. J.; Yazyev O. V. Moiré Flat Bands in Twisted Double Bilayer Graphene. Nano Lett. 2020, 20, 2410–2415. 10.1021/acs.nanolett.9b05117. 32097013

[ref49] Burg G. W.; et al. Correlated Insulating States in Twisted Double Bilayer Graphene. Phys. Rev. Lett. 2019, 123, 197702 10.1103/PhysRevLett.123.197702. 31765206

[ref50] Lee J. Y.; et al. Theory of correlated insulating behaviour and spin-triplet superconductivity in twisted double bilayer graphene. Nat. Commun. 2019, 10, 5333 10.1038/s41467-019-12981-1. 31767862 PMC6877569

[ref51] Koshino M. Band structure and topological properties of twisted double bilayer graphene. Phys. Rev. B 2019, 99, 235406 10.1103/PhysRevB.99.235406.

[ref52] Slizovskiy S.; et al. Out-of-Plane Dielectric Susceptibility of Graphene in Twistronic and Bernal Bilayers. Nano Lett. 2021, 21, 6678–6683. 10.1021/acs.nanolett.1c02211. 34296602 PMC8361429

[ref53] Zhang Y.; et al. Direct observation of a widely tunable bandgap in bilayer graphene. Nature 2009, 459, 820–823. 10.1038/nature08105. 19516337

[ref54] McCann E. Asymmetry gap in the electronic band structure of bilayer graphene. Phys. Rev. B 2006, 74, 161403 10.1103/PhysRevB.74.161403.

[ref55] Laturia A.; Van de Put M. L.; Vandenberghe W. G. Dielectric properties of hexagonal boron nitride and transition metal dichalcogenides: from monolayer to bulk. NPJ. 2D Mater. Appl. 2018, 2, 6 10.1038/s41699-018-0050-x.

