# Supplementary material for: ARPES Signatures of Few-Layer Twistronic Graphenes
Source: Nano Lett. 2023 May 26;23(11):5201–8. doi: 10.1021/acs.nanolett.3c01173 (PMC10273478; doi:10.1021/acs.nanolett.3c01173)
Supplement: Supplementary file 1 — nl3c01173_si_001.pdf [file nl3c01173_si_001.pdf]

Supplementary Information: ARPES signatures of few-layer twistronic graphenes

James E. Nunn[†1,2], Andrew McEllistrim[†3,4], Astrid Weston[†3,4], Aitor Garcia-Ruiz[3,4], Matthew D. Watson[1], Marcin Mucha-Kruczynski[5], Cephise Cacho*[1], Roman V. Gorbachev*[3,4], Vladimir I. Fal'ko*[3,4], and Neil R. Wilson*[2]

[1]*Diamond Light Source, Division of Science, Didcot, OX11 0DE, UK*
[2]*Department of Physics, University of Warwick, Coventry CV4 7AL, UK*
[3]*School of Physics and Astronomy, University of Manchester, Oxford Road, Manchester M13 9PL, UK*
[4]*National Graphene Institute, University of Manchester, Booth St East, Manchester M13 9PL, UK*
[5]*Centre for Nanoscience and Nanotechnology, Department of Physics, University of Bath, Bath, BA2 7AY, UK*

[†] These authors contributed equally to this work. * corresponding authors e-mail: Vladimir.Falko@Manchester.ac.uk, Roman@Manchester.ac.uk, Cephise.Cacho@Diamond.ac.uk and Neil.Wilson@Warwick.ac.uk

Contents

1 Summary of Methods 2

2 Sample fabrication 3

3 μARPES 4

4 Continuum model for twisted few-layer graphene 5

5 Simulating ARPES intensity 7

6 Twist angle determination 10

7 Associating spectral features with electronic bands 10

8 Analysis of replica band intensity 11

9 EDC analysis of hybridisation gaps 12

10 tDBG flatband 13

11 Self-consistent analysis of the effect of a back-gate voltage 14

12 Analysis of gated tMBG Dirac cones 15

1 Summary of Methods

Sample fabrication

Samples were fabricated in an argon atmosphere using a modified PMMA-based tear-and-stack technique [1], controlled by a remote micromanipulation rig. Pre-prepared hBN on graphite flakes (on SiO_2 substrates) were used as an adhesive layer to tear the graphene flakes supported on a PMMA membrane, allowing accurate control over the twist angle. Samples were annealed in UHV at 300°C for several hours prior to measurement. For further details see SI section 2.

Angle resolved photoemission spectroscopy

ARPES experiments were performed at the nanoARPES branch of the I05 beamline of Diamond Light Source. A choice of two focusing optics are available to perform spatially resolved ARPES: a Fresnel zone plate for submicrometre spatial resolution, and a capillary mirror for improved flux and energy resolution (∼4 µm spatial resolution). All photoemission spectra in the main text were measured using the capillary mirror, apart from the tMBG data in the second panel of Fig. 2e which was measured with the zone plate, with a 90 eV photon energy and linearly polarised light, at a measured sample temperature of < 85 K. Experimental constant energy cuts were averaged over ±5 meV of the stated energy. For further details see SI section 3.

Tight-binding modelling of electronic structure A hybrid $\mathbf{k}\cdot\mathbf{p}$ theory-tight-binding Hamiltonian was used for the twisted structures, as previously reported [2–4]. The SWMcC parameters used for this description are shown in Table 1 and are taken from [5]. Further details are given in SI section 4.

γ_0 (eV)	γ_1 (eV)	γ_3 (eV)	γ_4 (eV)	$\Delta'(eV)$
3.16	0.39	0.315	0.07	0.025

Table 1: SWMcC parameters, as in [5].

ARPES simulations. The general form of the ARPES intensity in the central mBZ is written as:

$$I \propto |\langle\psi_{\text{vac}}|\nabla_{\mathbf{k}}H|\psi_{\text{band}}\rangle|^2\mathcal{L}(E_{\mathbf{p}} + W - \epsilon_{\mathbf{q}} - \hbar\omega)$$
$$\propto |\sum_{l=1}^{3}(c_{l,A}e^{i\theta_A} + c_{l,B}e^{i\theta_B}) * F^{l-1}|^2\mathcal{L}(E_{\mathbf{p}} + W - \epsilon_{\mathbf{q}} - \hbar\omega), \quad \text{(S1)}$$

where H is the twisted graphene Hamiltonian [6] (see SI section 4).The initial state, ψ_{band}, is the wavefunction of the graphene electron, written as a linear combination of Bloch functions at a given point in momentum space, comprised of layer and sublattice components that are coupled using SWMcC parameters as well as mixed by the moiré [7]. The components are solved by diagonalising the respective system Hamiltonian and solving for its wavefunctions. From this, the weights ($c_{l,\lambda}$) are computed for each lattice site, where l is the layer number and λ is the sub-lattice index. The final state, ψ_{vac}, is assumed to be a plane wave in the vacuum. The interaction term $\nabla_{\mathbf{k}}H$ adds a small phase shift to the ARPES spectra. Attenuation and interference after photoemission are accounted for by the term: $F = Ae^{ik_z\cdot d}$, where $A = 0.4$ per graphene layer was determined by comparison to experiment. $d = 3.35$ Å is the distance between adjacent layers and k_z is the out-of-plane component of the final state momentum, calculated by determining k_z from the conservation of energy ($E_p + W = \epsilon_q - \hbar\omega$) and the kinetic energy of an emitted photoelectron ($E_p = \hbar^2(k_z^2 + k_\parallel^2)/2m_e$). The Lorentzian factor $\mathcal{L}(E_{\mathbf{p}} + W - \epsilon_{\mathbf{q}} - \hbar\omega)$ broadens the spectra to match the experimental broadening of 60 meV. Further details are given in SI section 5.

2 Sample fabrication

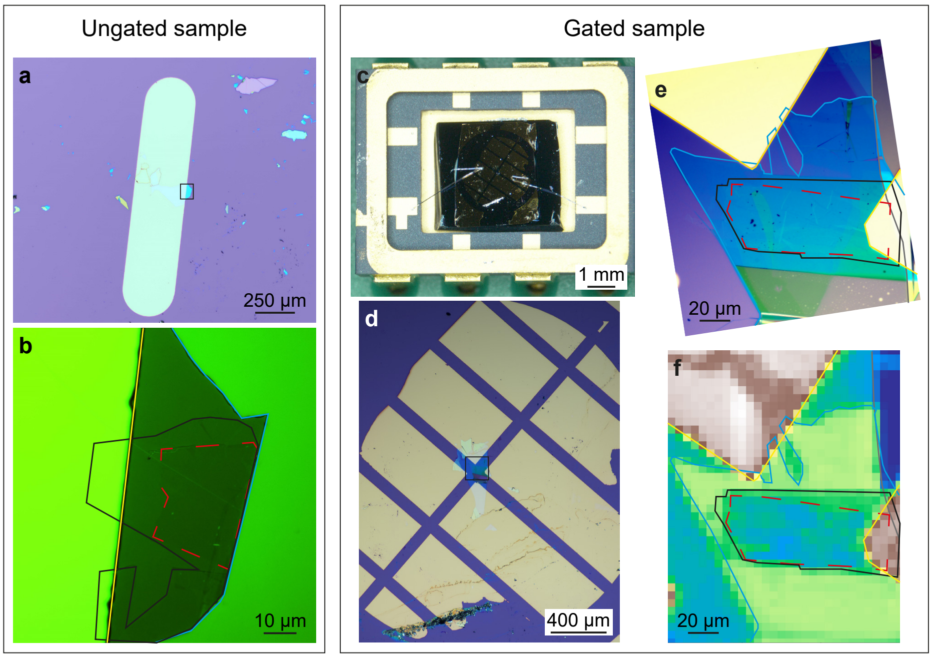

Figure S1: Sample summary. **a** Low magnification optical microscope image of a sample with a single, grounding contact (ungated sample). The region of interest (black box) is contacted by a large Au contact for grounding. **b** Higher magnification optical microscope image from the black box region in **a**. A green filter has been used to enhance the image contrast. The coloured outlines mark the Au contact (yellow), hBN (blue) and graphene (black) regions. The red dashed line marks the twisted graphene region. **c** Photograph of a chip carrier mounted with a sample with separate electrical contacts to graphene and graphite back-gate (gated sample), with wire bonds between sample contacts and chip carrier pads. **d** Low magnification microscope image of the gated sample in **c**. **e** Higher magnification microscope image from the black box region in **d**. **f** SPEM image of the sample region in **e**. The graphene (black), Au contacts (yellow), hBN (blue) and bottom graphite electrode (grey) sections can all be easily distinguished from each other within the SPEM image, allowing mapping of the sample while in the ARPES chamber.

Sample fabrication was performed using a remotely controlled micromanipulation rig housed inside an argon atmosphere. Twisted multilayers of graphene (including tBG, tMBG and tDBG) were transferred onto hBN/Si(SiO_2-290nm) and hBN/graphite/Si(SiO_2-290nm) stacks for ungated and gated samples respectively. The hBN/graphite heterostructures were prepared using a standard PMMA-based dry transfer technique [1], including mechanical exfoliation of crystals on silicon coated with a sacrificial poly-vinyl alcohol (PVA) layer as well as a poly-methyl methacrylate (PMMA) carrier layer. For the twisted graphene multilayers, we employed a modified tear-and-stack technique [8] to the PMMA-based dry transfer method to manipulate the twist angle between the graphene layers. The graphene was placed over the edge of the hBN (with the other half touching the SiO_2/Si and avoiding the graphite for the gated samples) to tear the graphene layer in half. The sample was then rotated by a target twist angle, θ, before stacking the 2nd half of the graphene layer on top of the first. A single flake of monolayer or bilayer graphene was used for tBG and tDBG twisted samples, respectively. For tMBG twisted samples, flakes consisting of a monolayer attached to bilayer regions were used. The top twisted graphene layer (and bottom graphite flake for gated samples) were contacted using Ti (3 nm)/Au (40 nm) electrodes

deposited through a TEM grid shadow mask to minimise contamination. Optical images of ungated and gated samples are shown in Fig. S1. Gated samples were mounted into chip carriers (Fig. S1c) using a room temperature curing, UHV compatible, non-conductive epoxy purchased from Atom Adhesives (AA-bond 2116). Electrical connections between the chip carrier and sample electrodes were made using a wire bonder.

Samples were transferred to the beamline in air. Prior to measurement, samples were annealed in UHV at 300°C for several hours to remove surface adsorbates. Samples without gate electrodes were annealed for ∼3 hours, while samples mounted in the chip carriers were annealed for at least 6 hours.

3 μARPES

ARPES experiments were performed at the nanoARPES branch of the I05 beamline of Diamond Light Source. Here, synchrotron light is focused to a small spot size using specialised optics to allow for high spatial resolution ARPES. A choice of two focusing optics are available: a Fresnel zone plate for submicrometre spatial resolution, and a capillary mirror for improved flux and energy resolution (∼4 µm spatial resolution). All photoemission spectra in the main text were measured using the capillary mirror (apart from the bilayer on monolayer graphene data in the second panel of Fig. 2e with a 90 eV photon energy and linear horizontal polarised light. Data was collected using a Scienta Omicron DA30 hemispherical analyser, with a chamber pressure of 1 x 10^{-10} mbar and a measured sample temperature <85 K.

The sample was located and mapped in the ARPES chamber using scanning photoemission microscopy (SPEM). In SPEM, the sample was raster scanned under the focused beam whilst collecting an $I(E, k)$ spectrum at each position, where the direction of k is determined by the orientation of the analyser relative to the sample, giving a 4D dataset of $I(E, k, x, y)$. Each pixel of the SPEM image shown in Fig. S1f is an integrated intensity of the $I(E, k)$ spectrum from that point on the sample. By comparing this with optical images collected during fabrication, different regions on the sample can be easily identified. **k**-space mapping was performed by rotating the chamber, including the analyser, around the fixed sample and optic, to measure from different polar emission angles. Electrostatic gating measurements were performed using a Keithley 2634B Source Meter, applying a voltage to the graphite back gate layer, while a separate ground contact connects to the twisted graphene layers adjacent to the measurement position (e.g. Fig. S1e). Achieving an effective gate *in-situ* requires good electrical isolation between the two contacts, requiring precise alignment of the contact pads relative to the flakes as well as careful handling. It was found that not all the samples fabricated with gates could be electrically biased *in-situ*.

Samples of twisted graphene fabricated by exfoliation and mechanical stacking are known to show heterogeneity at the micrometre scale, with domains of different angular orientation separated by wrinkles, bubbles, and, sometimes, regions displaying commensurate to incommensurate transitions [9].

To assess the uniformity of our samples, we conducted AFM measurements and SPEM mapping prior to acquiring detailed spectra from specific positions. This is summarised in Fig. S2 for a tDBG sample. From the AFM images, uniform areas of interest a few micrometres across can be identified that are free from wrinkles and bubbles (Fig. S2b). On some samples, AFM brushing ('nano-squeegee' [10]) was used in specific locations to remove contamination between the layers and further improve the uniformity, as indicated by the arrows in Fig. S2b. SPEM mapping was used to locate these regions for ARPES analysis.

By integrating over a selected area of energy and momentum in the $I(E, k)$ spectra at each point on the sample, Fig. S2h(i) red dashed boxes, different flakes can be highlighted in the SPEM images (Fig. S2d,e). Comparison with optical and AFM images allows determination of the areas of interest. Averaging the $I(E, k, x, y)$ dataset within these positions indeed shows them to be uniform - sharp bands and clear hybridisation gaps are maintained (Fig. S2f,g). To further demonstrate this, a series of ARPES spectra from different positions are shown in Fig. S2h. When in a heterogeneous area, the spectra appear broadened, and contributions from different twist domains gives a superposition of bands with no clear interactions. By applying this simple spatial analysis, we could identify uniform areas of different twist angle within our samples and identify regions of different twist angles on a given sample. The data

presented in the main text come from 17 different measurements ($I(E, k_x, k_y)$ spectra from different twist-angles and/or stacking geometries) which were acquired from 9 different samples (4 tBG samples, 4 tMBG samples, 1 tDBG sample).

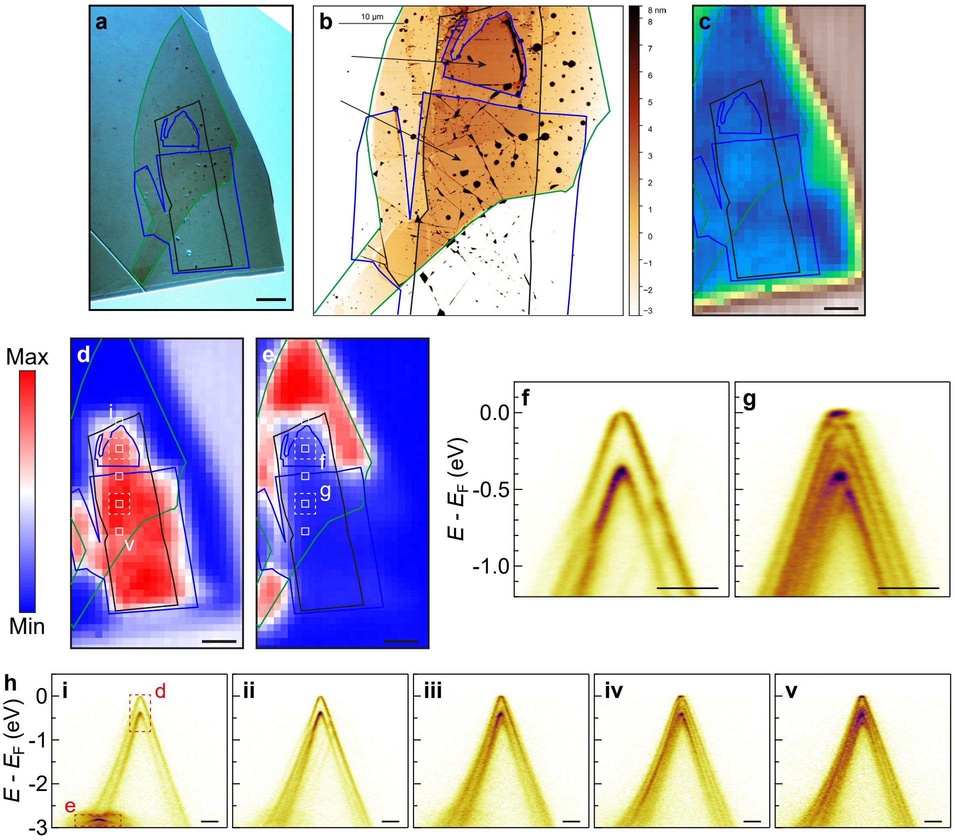

Figure S2: Sample mapping and uniformity determined by SPEM. **a-c** Optical, AFM and SPEM images of a tDBG sample, respectively. The top bilayer (black), bottom bilayer (blue) and hBN (green) flakes have been outlined. Arrows in **b** mark AFM brushed regions. **d,e** SPEM images integrated over the red dashed areas in **h(i)**. Real-space scale bars are 10 µm. **f,g** Total ARPES spectra acquired by summing over the individual ARPES spectra from the white bashed boxes in **d,e**. **h** ARPES spectra from the SPEM image positions marked by the white boxes in **d,e**. Reciprocal-space scale bars are 0.2 Å^{-1}.

4 Continuum model for twisted few-layer graphene

For bilayer graphene (BLG), the standard Hamiltonian as defined by McCann et al. [11] was used. For each twisted structure, a hybrid **k**·**p**-tight binding Hamiltonian was used for the twisted structures, similar to those previously reported [2–4]. Such Hamiltonians consider the dispersion around a given valley in reciprocal space and information can be obtained about the system by solving for the eigenfunctions and eigenvalues of the system. The Hamiltonians for twisted bilayer graphene (tBG), twisted monolayer-bilayer graphene (tMBG) and twisted double-bilayer graphene (tDBG), respectively are given below:

$$\mathcal{H}_{tBG} = \begin{pmatrix} 0 & v\hbar\pi^{\dagger}_{\xi,t} & T_{11} & \mathcal{T}_{12} \\ v\hbar\pi_{\xi,t} & 0 & \mathcal{T}_{21} & \mathcal{T}_{22} \\ \mathcal{T}^{\dagger}_{11} & \mathcal{T}^{\dagger}_{21} & 0 & v\hbar\pi_{\xi,b} \\ \mathcal{T}^{\dagger}_{12} & \mathcal{T}^{\dagger}_{22} & v\hbar\pi_{\xi,b} & 0 \end{pmatrix}, \tag{S2}$$

$$\mathcal{H}_{tMBG} = \begin{pmatrix} 0 & v\hbar\pi^{\dagger}_{\xi,t} & \mathcal{T}_{11} & \mathcal{T}_{12} & 0 & 0 \\ v\hbar\pi_{\xi,t} & 0 & \mathcal{T}_{21} & \mathcal{T}_{22} & 0 & 0 \\ \mathcal{T}^{\dagger}_{11} & \mathcal{T}^{\dagger}_{21} & 0 & v\hbar\pi_{\xi,b} & -v_4\hbar\pi^{\dagger}_{\xi,b} & -v_3\hbar\pi_{\xi,b} \\ \mathcal{T}^{\dagger}_{12} & \mathcal{T}^{\dagger}_{22} & v\hbar\pi_{\xi,b} & \Delta' & \gamma_1 & -v_4\hbar\pi^{\dagger}_{\xi,b} \\ 0 & 0 & -v_4\hbar\pi_{\xi,b} & \gamma_1 & \Delta' & v\hbar\pi_{\xi,b} \\ 0 & 0 & -v_3\hbar\pi^{\dagger}_{\xi,b} & -v_4\hbar\pi_{\xi,b} & v\hbar\pi_{\xi,b} & 0 \end{pmatrix}, \tag{S3}$$

$$\mathcal{H}_{tDBG} = \begin{pmatrix} 0 & v\hbar\pi^{\dagger}_{\xi,t} & -v_4\hbar\pi^{\dagger}_{\xi,t} & -v_3\hbar\pi_{\xi,t} & 0 & 0 & 0 & 0 \\ v\hbar\pi_{\xi,t} & \Delta' & \gamma_1 & -v_4\hbar\pi^{\dagger}_{\xi,t} & 0 & 0 & 0 & 0 \\ -v_4\hbar\pi_{\xi,t} & \gamma_1 & \Delta' & v\hbar\pi_{\xi,t} & \mathcal{T}_{11} & \mathcal{T}_{12} & 0 & 0 \\ -v_3\hbar\pi^{\dagger}_{\xi,t} & -v_4\hbar\pi_{\xi,t} & v\hbar\pi_{\xi,t} & 0 & \mathcal{T}_{21} & \mathcal{T}_{22} & 0 & 0 \\ 0 & 0 & \mathcal{T}^{\dagger}_{11} & \mathcal{T}^{\dagger}_{21} & 0 & v\hbar\pi_{\xi,b} & -v_4\hbar\pi^{\dagger}_{\xi,b} & -v_3\hbar\pi_{\xi,b} \\ 0 & 0 & \mathcal{T}^{\dagger}_{12} & \mathcal{T}^{\dagger}_{22} & v\hbar\pi_{\xi,b} & \Delta' & \gamma_1 & -v_4\hbar\pi^{\dagger}_{\xi,b} \\ 0 & 0 & 0 & 0 & -v_4\hbar\pi_{\xi,b} & \gamma_1 & \Delta' & v\hbar\pi_{\xi,b} \\ 0 & 0 & 0 & 0 & -v_3\hbar\pi^{\dagger}_{\xi,b} & -v_4\hbar\pi_{\xi,b} & v\hbar\pi_{\xi,b} & 0 \end{pmatrix}. \tag{S4}$$

$\pi_{\xi,t/b}$ is defined as:

$$\pi_{\xi,t/b}(p) \approx -\frac{\sqrt{3}a}{2\hbar}(\xi p_x + i(p_y \mp K\theta/2)) + \frac{a^2}{8\hbar^2}(\xi p_x - i(p_y \mp K\theta/2))^2, \qquad K = \frac{4\pi}{3a}, \tag{S5}$$

where p_x, p_y are the in-plane components of the momentum shifted to be centred around the K_+ valley ($p = \hbar k - \hbar\kappa_+$, where $\kappa_+ = (4\pi/3a, 0)$). In Eq. (S5), a second-order expansion in momentum is used to account for trigonal warping of the band structure at higher energies. The interlayer coupling matrices $\mathcal{T}_{ij}$ across the twisted interface are defined as:

$$\mathcal{T}_{k',k} = \begin{pmatrix} \mathcal{T}_{11} & \mathcal{T}_{12} \\ \mathcal{T}_{21} & \mathcal{T}_{22} \end{pmatrix} = \frac{\gamma_1}{3}\sum_{j=0}^{2} \begin{pmatrix} 1 & e^{i\xi\frac{2\pi}{3}j} \\ e^{-i\xi\frac{2\pi}{3}j} & 1 \end{pmatrix} \delta_{\mathbf{k',k}+\Delta\mathbf{K}^{(0)}_{\xi}-\Delta\mathbf{K}^{(j)}_{\xi}} \tag{S6}$$

where $\Delta\mathbf{K}^{(j)}_{\xi} = \theta(\mathbf{K}^{(j)}_{\xi,x} - \mathbf{K}^{(j)}_{\xi,y})$ and $K^{(j)}_{\xi} = K[\xi\cos(2\pi j/3), -\sin(2\pi j/3)]$, with j = 0, 1, 2 and $K = 4\pi/3a$.

Table 2 gives the values for the Slonczewski-Weiss-McClure (SWMcC) parameters used for all of the calculations here.

v (m/s)	γ_1 (eV)	v_3 (m/s)	v_4 (m/s)	Δ' (eV)
1.02 x 10^6	0.39	1.02 x 10^5	2.27 x 10^4	0.025

Table 2: The SWMcC parameters used here, with values taken from [5].

where $v_i = \sqrt{3}a\gamma_i/2\hbar$. v accounts for the intralayer coupling between the A and B sites in the graphene lattices. γ_1 corresponds to the interlayer coupling between the dimer sites (A_b/B_t) of the aligned bilayers and is also used to compute the interlayer coupling at the twisted interfaces. v_3, v_4

account for the coupling between the non-dimer sites and the coupling between a dimer site and a non-dimer site, respectively. Lastly, Δ' accounts for the difference in the on-site electron energies of the dimer sites. v_4 and Δ' also have the effect of adding electron-hole asymmetry into the optical response of the system.

5 Simulating ARPES intensity

The ARPES intensity is proportional to the square of the modulus of the transition amplitude between the initial and final states of the system under the perturbation caused by incoming photons [12, 13]. We define the initial state of the system as a single Bloch electron at the surface. The general form of a Bloch wave is:

$$\Phi_j(r) = \sum_j C_j \varphi_{k,j}(\mathbf{r}) \approx \sum_j \sum_{\lambda=A,B} (c_{j,\lambda} e^{i\theta_\lambda}) e^{i\mathbf{G}_m \cdot \mathbf{r}}, \tag{S7}$$

where j is the band index of the system and C_j is the amplitude of the wavefunction of the system, containing a sublattice phase. The wavefunctions, $\varphi_{k,j}(\mathbf{r})$, are calculated by diagonalising the Hamiltonians shown in Eqs. (S2),(S3) and (S4). $\mathbf{G}_m$ are the moiré reciprocal lattice vectors. The final state of the system, the emitted photoelectron with momentum $\mathbf{p}_e$, is treated as a plane wave with approximate form:

$$\Phi_f(r) \propto \exp\left(\frac{i}{\hbar}\mathbf{p}_e \cdot \mathbf{r}\right) \approx \mathbb{1}. \tag{S8}$$

The perturbation due to the incoming radiation is treated as a first-order approximation of the standard perturbative Hamiltonian:

$$\begin{aligned} \mathcal{H}_p &= -\frac{2}{2m_e}(\mathbf{A}\cdot\mathbf{v} + \mathbf{v}\cdot\mathbf{A}) = -\frac{e}{\hbar c}\mathbf{A}\cdot\mathbf{v} \\ &= -\frac{e}{\hbar c}\mathbf{A}\cdot\nabla_k H, \end{aligned} \tag{S9}$$

where $\mathbf{A}$ is the electromagnetic vector potential of the incoming radiation, H is the Hamiltonian of the graphene system, and $\mathbf{v}$ is the electron velocity operator which introduces the interaction Hamiltonian ($H_{int} = \nabla_k H$) for the photoemission process.

Accounting for multi-layer effects, the general form of the ARPES intensity in the central mini-Brillouin zone can then be written as:

$$\begin{aligned} I &\propto |< \varphi_f|\mathbf{A}\cdot\mathbf{v}|\varphi_i >|^2 \, \delta(\epsilon_e + W - \epsilon_M - \hbar\omega) \\ &= |\mathbf{A}\cdot < \varphi_f|\nabla_k H|\varphi_i >|^2 \, \delta(\epsilon_e + W - \epsilon_M - \hbar\omega) \\ &\propto |\sum_{l=1}^{3}(c_{l,A}e^{i\theta_A} + c_{l,B}e^{i\theta_B}) * F^{l-1}|^2 \mathcal{L}(\epsilon_e + W - \epsilon_M - \hbar\omega), \end{aligned} \tag{S10}$$

where $\hbar\omega$ is the energy of the photons used in the experiment, $W = 4.6$ eV is the workfunction of graphene [14], and ϵ_M is the energy of the measured electron in the crystal lattice. The delta function comes from treating a single electron in a many-body system using a time-ordered one-electron Green's function [15]. To give a qualitative match to the experimental spectra, accounting for instrument resolution and lifetime broadening etc., a Lorentzian with 60 meV broadening is included ($\mathcal{L}(\epsilon_e + W - \epsilon_M - \hbar\omega)$). $\mathbf{A}$ is a 2x1 vector whose value can be changed to alter the polarisation of the light in the system. Eq. (S10) thus gives an analytical approximation to the ARPES intensity.

Accounting for attenuation and interference across multi-layers We do not attempt a one-step photoemission model [15], but instead account for attenuation and interference of the photo-emitted electrons from different layers using a scaling factor, F, assuming a plane wave final state. This scaling factor accounts for attenuation (due to the short mean free path of photo-electrons) and interference

(due to layer-dependent phase differences of the photo-electrons): $F = Ae^{ik_z \cdot d}$, where k_z is the out-of-plane momentum of the photo-emitted electrons and $d = 3.35$ Å is the distance between adjacent graphene layers. The attenuation factor here was set to be $A = 0.4$ per layer, found by comparison to the experimental data. Starting with the kinetic energy of the emitted photo-electron

$$E_p = \hbar^2 \frac{k_z^2 + K_\parallel^2}{2m_e}, \tag{S11}$$

using conservation of energy, the out-of-plane momentum is

$$k_z = \sqrt{\frac{2m_e}{\hbar^2}\left[\hbar\omega - W + \epsilon_M - \frac{\hbar^2}{2m_e}\left[\left(\frac{4\pi}{3a} + p_x\right)^2 + p_y^2\right]\right]}. \tag{S12}$$

This defines all terms in the factor $F^{l-1} = (Ae^{ik_z \cdot d})^{l-1}$ seen at the end of Eq. (S10), where l is the layer number counting from the top surface.

As a consequence of Eq. (S12), the phase difference between photoelectrons emitted from different layers depends on the energy of the incident photon used. This phase factor is plotted as a function of the photon energy in Fig. S3, for photoelectrons emitted from neighbouring graphene layers, across the experimentally relevant photon energies. Note that the phase factor changes significantly with photon energy, indicating that the relative intensity of different features in the ARPES spectra should be strongly dependent on the photon energy. The validity of this approach was tested by comparing experimental and predicted spectra for bilayer graphene and tMBG at varying photon energy, as shown in Fig. S4. While many of the key features are consistent across the different energies, the relative intensities of features changes with photon energy. The results show that although this simple model is not a full description of the photoemission process, it gives a good approximation. We also note that the elongated flat region at the beginning of Fig. S3 corresponds to the fact that it takes $\epsilon = W$ to photoexcite electrons from the Γ point but additional energy to photoexcite electrons with nonzero in-plane momentum.

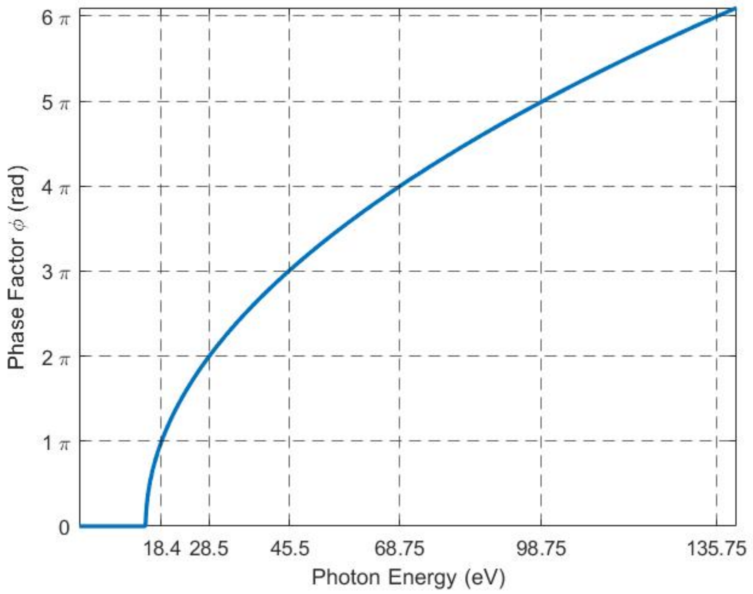

Figure S3: Approximate phase factor, $\phi = k_z \cdot d$, as a function of photon energy.

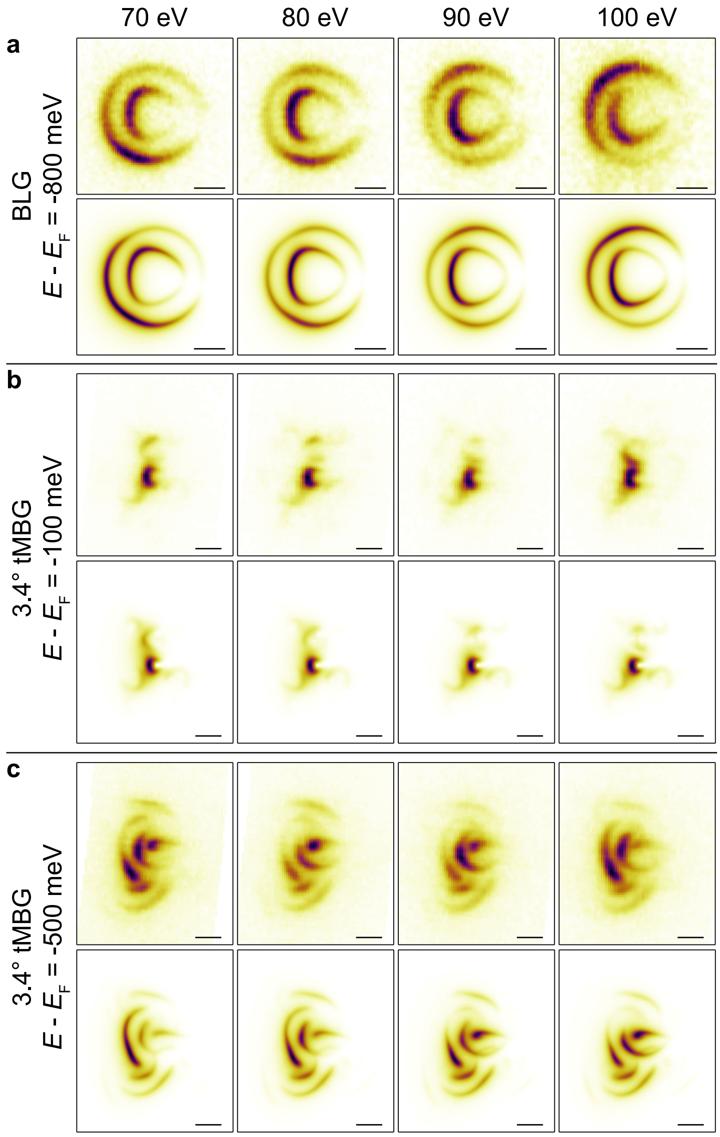

Figure S4: Changes in the ARPES spectra of multilayered graphene systems due to varying photon energy. Constant energy cuts for **a** bilayer graphene at $E - E_{\mathrm{F}}$ = -800 ± 15 meV, **b** 3.4° tMBG at $E - E_{\mathrm{F}}$ = -100 ± 5 meV and **c** 3.4° tMBG at $E - E_{\mathrm{F}}$ = -500 ± 5 meV at photon energies (moving left to right) 70 eV, 80 eV, 90 eV and 100 eV. Top panels are from experimental ARPES spectra, while bottom panels are from simulation. All scale bars are 0.1 Å^{-1}.

We note that the comparison between simulated and experimental spectra in Fig. S4 can also be used to confirm the sign of the tight binding parameter γ_1. There is no clear consensus on the sign of γ_1 in the literature: in some reports it is assumed to be positive, and in others, negative [12]. In other reports, γ_1 is taken to be negative with a phase factor of $e^{i\pi}$ per layer replacing the factor $\exp(ik_z \cdot d)$ in F [16]. A comparison of simulated and experimental spectra demonstrates that the sign of γ_1 is positive with no additional phase needed.

6 Twist angle determination

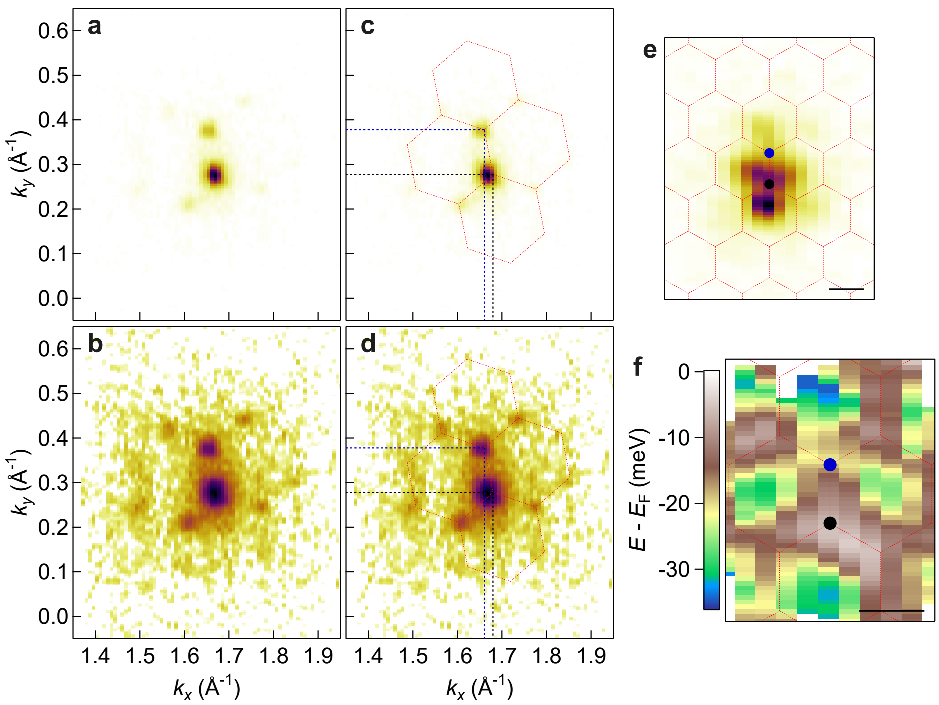

Figure S5: Determining the twist angle of twisted graphene from ARPES. **a,b** Constant energy cuts for 3.4° tBG at $E - E_F = 0$ displayed on a linear and logarithmic scale, respectively, averaged over ±15 meV of the specified energy. **c,d** The same constant energy cut as in **a** and **b** overlaid with four mBZs and dashed lines to mark the coordinates of the κ_1 and κ_2 positions. **e,f** Constant energy cut at $E - E_F = 30$ meV and extracted flat band energy of 1.5° tDBG, respectively, as described in Fig. 3 of the main text. Scale bars are 0.05 Å^{-1}.

We determined the twist angles directly from the ARPES spectra. Replicas bands at E_F are positioned at the mBZ corners, which form a honeycomb pattern with the primary bands (Fig. S5a,b). These periodic features were used to determine the mBZs, which provide the κ_1 and κ_2 positions. The twist angle can then be calculated from

$$|\kappa_2 - \kappa_1| = \frac{8\pi}{3a} \sin\left(\frac{\theta}{2}\right). \tag{S13}$$

The error on the twist angle was estimated from the uncertainty in positioning the mBZs due to the finite width of primary and replica bands at E_F.

For the case of 1.5° tDBG, with the flat band at E_F replicas are not readily resolved at the mBZ corners. Instead, we used the periodicity of the intensity at γ to position the mBZ, both in the constant energy cuts (Fig. S5e) and the dispersion of the flat band (Fig. S5f). Again, the error bar on this comes from the uncertainty in positioning the mBZs.

7 Associating spectral features with electronic bands

For small-twist angle samples at higher binding energies, the spectra become more complex, as shown in Figs. S6a,b, and it becomes difficult to distinguish which layer and band each spectral feature is associated with. Comparison between the simulated and experimental spectra, alongside the band structure

predictions, enables the ARPES spectral features to be assigned to distinct valence bands. For example, in Fig. S6, constant energy cuts of the ARPES spectra are shown alongside the band structure in the $k_x - k_y$ plane averaged over the same energy window, $E \pm 30$ meV, shown in the extended zone scheme such that the pattern is repeated across successive mBZs. In Figs. S6e,f the bands that are most apparent in the ARPES spectra are highlighted. In the band calculation the colour scheme is as follows: blue, green, red, cyan = 1st, 2nd, 3rd, 4th valence band.

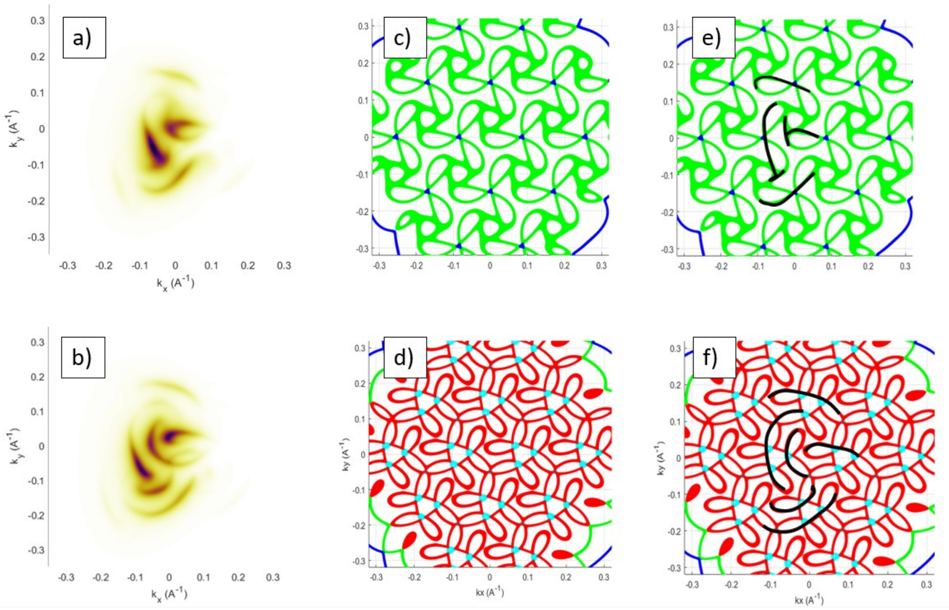

Figure S6: Identifying the band contributions in ARPES from tMBG. Simulated ARPES contant energy cuts at $E - E_F$ = **a** -350 meV and **b** -500 meV for 3.4° tMBG. **c,d** band structure of 3.4° tMBG at the same energies as in **a** and **b**, respectively. **e,f** Same as **c** and **d** overlaid with black lines to mark the bands contributing to the ARPES intensity in **a** and **b**.

8 Analysis of replica band intensity

As shown in Section 7, in the extended zone scheme, bands are equivalent in each mBZ. However, the photoemission intensity of the replica bands decreases in successive mBZs due to the reduced probability of scattering to higher wave vectors. Fig. S7a shows the photoemission intensity near the Fermi level for a 3.0° tBG. The primary Dirac points are labelled following the convention from the main text of κ_1 for the top layer and κ_2 for the bottom layer, where these now refer to an intensity. The photoemission intensity from the bottom layer is reduced relative to the top layer due to attenuation, i.e. $\kappa_2 = A \times \kappa_1$, where A is the previously mentioned attenuation set to match the experiment as $A = 0.4$. Intensity due to replica bands can be seen at the corners of the mBZs. These are labelled by the order of their respective intensity, i.e. R_1 is the most intense replica and R_{14} is the least intense. This is shown more clearly in Fig. S7b, where the total intensity is taken from the circular regions marked in Fig. S7a, and normalised with respect to the intensity at κ_1. This is compared to the experimental results where there is relatively good agreement and they roughly follow the same hierarchy.

The intensity ordering of the replicas is nontrivial. For example, we can associate replicas R_1 and R_2 with states from the bottom layer, and replicas R_3 and R_4 with states from the top layer. We know this because, in the case of tMBG, R_1 and R_2 show a bilayer graphene-like dispersion, while R_3 and R_4 show a monolayer graphene-like dispersion. Despite this, R_1 and R_2 show a greater intensity than R_3 and R_4,

even though states belonging to the bottom layer would be expected to be attenuated. In addition to this, replicas which we would expect to be equivalent with respect to their scattering distance from their respective primary show differing intensity. This is seen most clearly for the replicas R_1, R_2, R_7, R_9, R_{12} and R_{14}. Although they are all a single moiré reciprocal lattice vector away from κ_2, R_7, R_9, R_{12} and R_{14} are all at least an order of magnitude weaker than R_1 and R_2. The good agreement between experiment and theory confirms the validity of the model used for simulating the photoemission intensity.

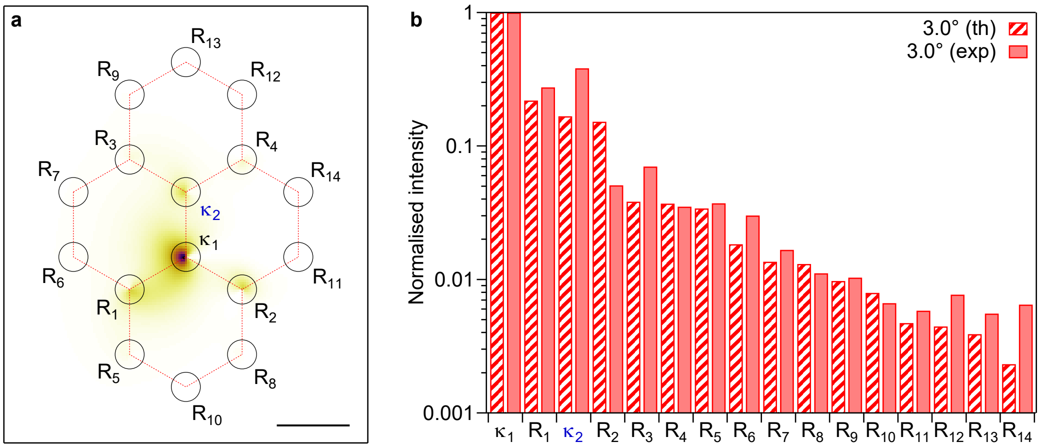

Figure S7: Analysis of replica band intensity in 3.0° tBG. **a** Simulated constant energy cut at the Fermi level of 3.0° tBG overlaid with the mBZ and labels for each mBZ corner. Scale bar is 0.1 Å^{-1}. **b** Replica intensity of 3.0° tBG from **a**, normalised by the intensity of the primary band from the top layer, κ_1, from experiment and simulation. The contributed intensity comes from the circular areas in **a**.

9 EDC analysis of hybridisation gaps

EDCs extracted from cuts along the κ_1-κ_2 direction were used to determine the size of hybridisation gaps. Examples are shown in Fig. S8 for each of the different twisted graphene stacking arrangements discussed in the main text. Extracted EDCs are fit to a pair of Gaussian functions on a constant background (Fig. S8d-g). The difference between the Gaussian peak positions is interpreted as the hybridisation gap size, δ.

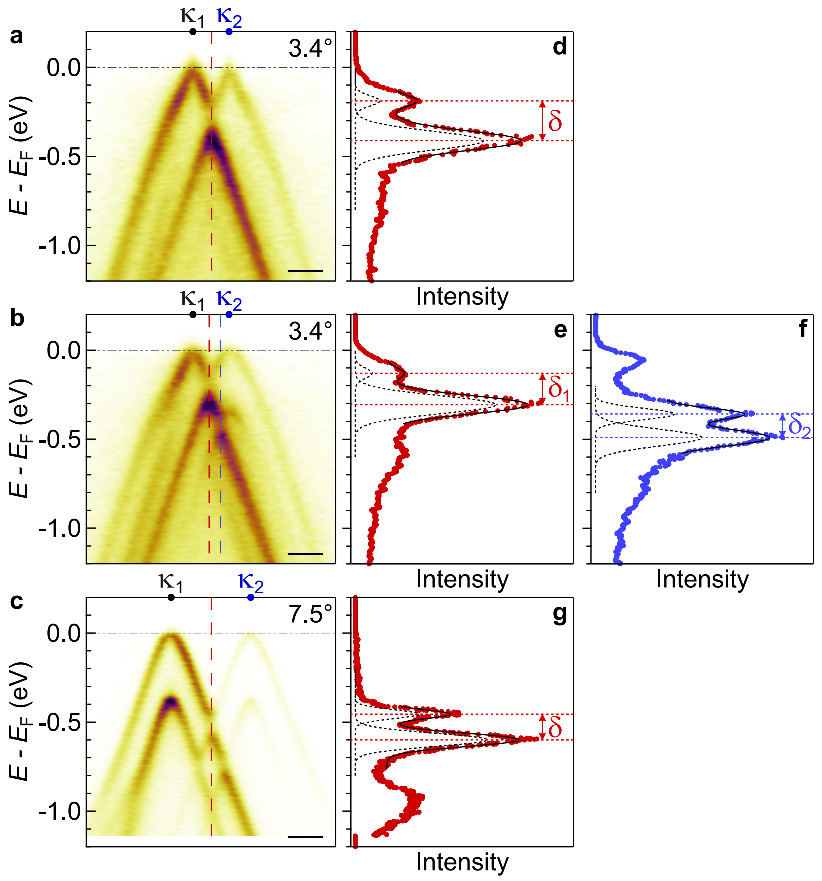

Figure S8: EDC analysis of hybridisation gaps. **a-c** ARPES spectra along the κ_1-κ_2 direction for tBG, tMBG and tDBG, respectively, at the specified twist angles. **d-g** EDCs extracted along the vertical dashed lines in **a-c** overlaid with a pair of Gaussians on a constant background fitting function. Dashed peaks show individual Gaussian fits, where the separation in their centres provides the hybridisation gap size, δ. All scale bars are 0.1 Å^{-1}.

10 tDBG flatband

Fig. S9 is an extended version of Fig. 3 from the main text including further comparison with the continuum model close to E_F. There are clear differences between the simulated ARPES spectra in the middle panels of Figs. S9a-c and the corresponding experimental spectra. Inspection of the band dispersions (right-hand panels) reveals that the gap between the upper valence band (the flat band) and the lower-lying valence bands is significantly smaller in the predicted dispersions (red lines) than the experimental band dispersions (black lines). Note that the 60 meV broadening applied to the simulated spectra in the main text is reduced to 40 meV here to match the improved quality of the experimental results attained for this device.

Fig S9d illustrates how the experimental band dispersions were obtained: the EDC is from the red dashed line in the left-hand panel of Fig S9a, the solid black line is a fit using two Gaussian peaks the positions of which give the band dispersions. In Fig. S9e, the energy of the flatband is plotted in the $k_x - k_y$ plane across the first few mBZs for both the continuum model calculated dispersion (left-hand panel) and the dispersion extracted from the experimental spectra (right-hand panel). Though the

features in the experimental data are broad, they agree with the prediction that the flat band minima should be at the γ point and show the expected periodicity across the mBZs.

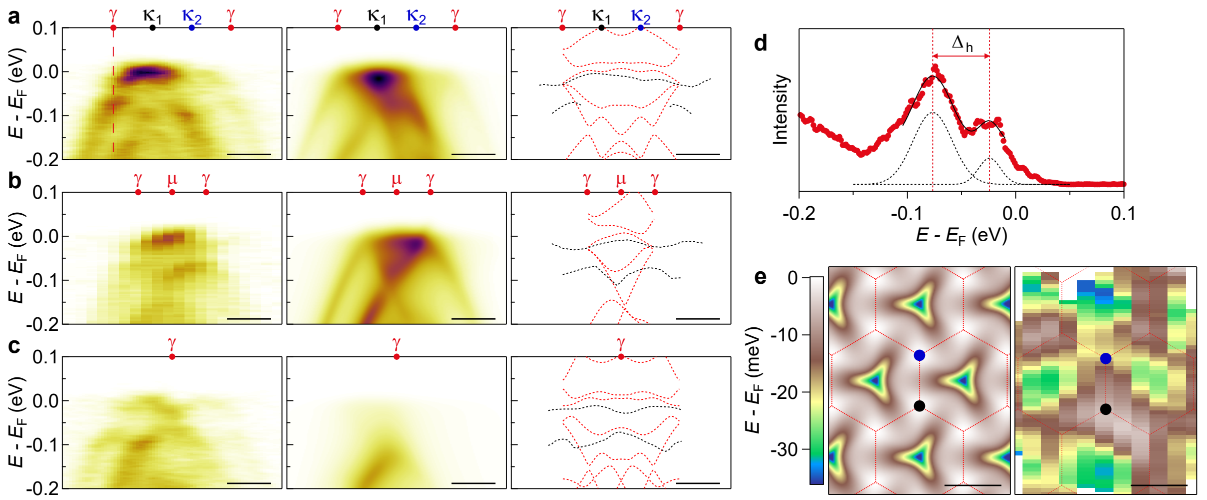

Figure S9: Simulated and experimental ARPES spectra of 1.5° tDBG. **a-c** Experimental (left-hand) and simulated (middle) energy-momentum cuts along the high symmetry directions, as in Fig. 3 of the main text, and the corresponding band dispersions (right-hand). The band dispersions are plotted in the reduced zone scheme of the mBZ. When comparing to the simulated spectra, it is important to recall that the primary bands of each BLG layer are intense across multiple mBZs whilst the intensities of replica bands decrease in higher mBZs and that the broadening of the spectra obscures small gaps and closely spaced bands. The black lines correspond to the peak positions extracted from the experimental data by fitting EDCs and the red lines correspond to the predicted electronic structure. **d** EDC along the vertical dashed line in the left-hand panel of **a**. The solid black line is a fit to the data with a pair of Gaussian peaks whose positions correspond to the band positions and separation gives the gap size, Δ_h. **e** Energy of the simulated (left-hand) and experimental (right-hand) flat-band plotted in the $k_x - k_y$ plane, with the mBZs overlaid in red. All scale bars are 0.05 Å^{-1}.

11 Self-consistent analysis of the effect of a back-gate voltage

The effect of a back gate voltage is included in the electronic structure model through a self-consistent analysis that accounts for the change in interlayer potential due to the displacement field and the resultant charge redistribution. The electric displacement field has the following form [4]:

$$D = \frac{V_G C_G}{2\epsilon_0}, \tag{S14}$$

where C_G is the capacitance to the back gate [17]. This can be used to calculate an initial interlayer potential [18], u, that is the difference between the potential on the top layer, U_t, and the bottom layer, U_b:

$$u_i = U_t - U_b = \frac{e c_0 D}{\epsilon_0 \epsilon_z}, \tag{S15}$$

where c_0 is the spacing between the layers and ϵ_z is the effective out-of-plane dielectric susceptibility. For tMBG, where there are three layers, the energy differences between the two outer layers and the inner

layer must be calculated. For this, as in [18], the following parameters are used:

$$\begin{aligned} c_{0,1} &= 3.44 \text{ Å for tBLG}, \\ c_{0,2} &= 3.35 \text{ Å for BLG}, \\ \epsilon_{z,1} &= 2.5 \text{ for tBLG}, \\ \epsilon_{z,2} &= 2.6 \text{ for BLG}. \end{aligned} \tag{S16}$$

The initial interlayer potential u_i is introduced to the Hamiltonian as follows:

$$H \longrightarrow H + \begin{pmatrix} 0 & 0 & 0 & 0 & 0 & 0 \\ 0 & 0 & 0 & 0 & 0 & 0 \\ 0 & 0 & -u_1 & 0 & 0 & 0 \\ 0 & 0 & 0 & -u_1 & 0 & 0 \\ 0 & 0 & 0 & 0 & -(u_1+u_2) & 0 \\ 0 & 0 & 0 & 0 & 0 & -(u_1+u_2) \end{pmatrix}. \tag{S17}$$

From the wavefunctions calculated using this new Hamiltonian, the new layer density n_i in each layer due to the back gate is calculated (n_t for the density in the upper monolayer graphene, n_m for the density in the middle layer, and n_b for the density in the bottom layer of the bilayer graphene), as are the energies of each layer:

$$n = \frac{\epsilon_0 \epsilon_{\text{hBN}} V_{\text{G}}}{d_{\text{hBN}} e}, \tag{S18}$$

$$n_i = 2 \int_{BZ} \frac{d^2k}{(2\pi)^2} \sum_{l=1}^{2N} \left[(|\Psi^l_{A_i}(k)|^2 + |\Psi^l_{B_i}(k)|^2) f(\epsilon_l - E_F) - \frac{1}{2} \right]. \tag{S19}$$

Here, $\epsilon_{\text{hBN}} = 4$ [19], and $d = 26$ nm is the thickness of the hBN layer for the data in Fig. 5 of the main text. In Eq. (S19), the index i denotes the layer and N is the number of bands being considered for the calculation. Ψ_λ are the wavefunctions and $f(\epsilon_l - E_{\text{F}})$ is the Fermi distribution. The wavefunctions are used to calculate a new set of interlayer energy differences and the calculations are iterated until they converge and the interlayer potentials are found self-consistently. For tMBG, these give the interlayer potential between the monolayer and the upper layer of the bilayer graphene, u_1, and between the upper and lower layers of the bilayer graphene, u_2:

$$\begin{aligned} u_1(D,n) &= \frac{eD_z c_{0,1}}{\epsilon_0 \epsilon_{z,1}} + \left[\frac{e^2(n_t - n_m)}{2\epsilon_0} \frac{1+\epsilon_{z,1}^{-1}}{2} - \frac{e^2 n_b}{2\epsilon_0 \epsilon_{z,1}} \right] c_{0,1} \\ u_2(D,n) &= \frac{eD_z c_{0,2}}{\epsilon_0 \epsilon_{z,2}} + \left[\frac{e^2(n_m - n_b)}{2\epsilon_0} \frac{1+\epsilon_{z,2}^{-1}}{2} + \frac{e^2 n_t}{2\epsilon_0 \epsilon_{z,2}} \right] c_{0,2} \end{aligned} \tag{S20}$$

Eq. (S19) is then used to calculate the layer densities shown in Fig. 5c of the main text.

12 Analysis of gated tMBG Dirac cones

To calculate the band parameters as a function of V_{G} from the experimental spectra, as plotted in Fig. 5 of the main text, energy momentum cuts through the κ_1-κ_2 direction were extracted for each gate voltage (Fig. S10a top panels). Momentum distribution curves (MDCs) were used to extract the band positions of the monolayer and bilayer cones close to the Dirac points. These were fit to Lorentzian functions on a constant background, with the peak centre providing the band position (Fig. S10b). Using standard low energy approximations to the electronic dispersions, the monolayer band positions were fit by $E = E_{\text{D}} - v|k - k_0|$, and the bilayer band positions by $E = E_{\text{D}} - \frac{1}{2}\gamma_1 \left[\sqrt{1 + 4v^2k^2/\gamma_1^2} - 1 \right]$,

Fig. S10a bottom panels, where v is a band velocity. The fitting coefficients provide the Dirac point energy E_D. From the tight-binding approximation to the isolated layers, the Dirac point energy can be used to calculate the carrier density, using expressions $n_\mathrm{MLG} = \frac{E_\mathrm{D}^2}{\pi v^2}$ and $n_\mathrm{BLG} = \frac{\gamma_1 E_\mathrm{D}}{\pi v^2}$ for the monolayer and bilayer, respectively, [20]. For simplicity, we have only fit to the valence band, and thus assume the monolayer and bilayer dispersions are symmetric about E_D.

The size of the gap at the Dirac point of the bilayer graphene, Δ, is measured in the same way as previously described for the hybridisation gaps, see section S7. An EDC is extracted through the centre of the bilayer cone and fit to a pair of Gaussian functions on a constant background (Fig. S10c). The gap can only be resolved for $V_\mathrm{G} \geq 7.5$ V.

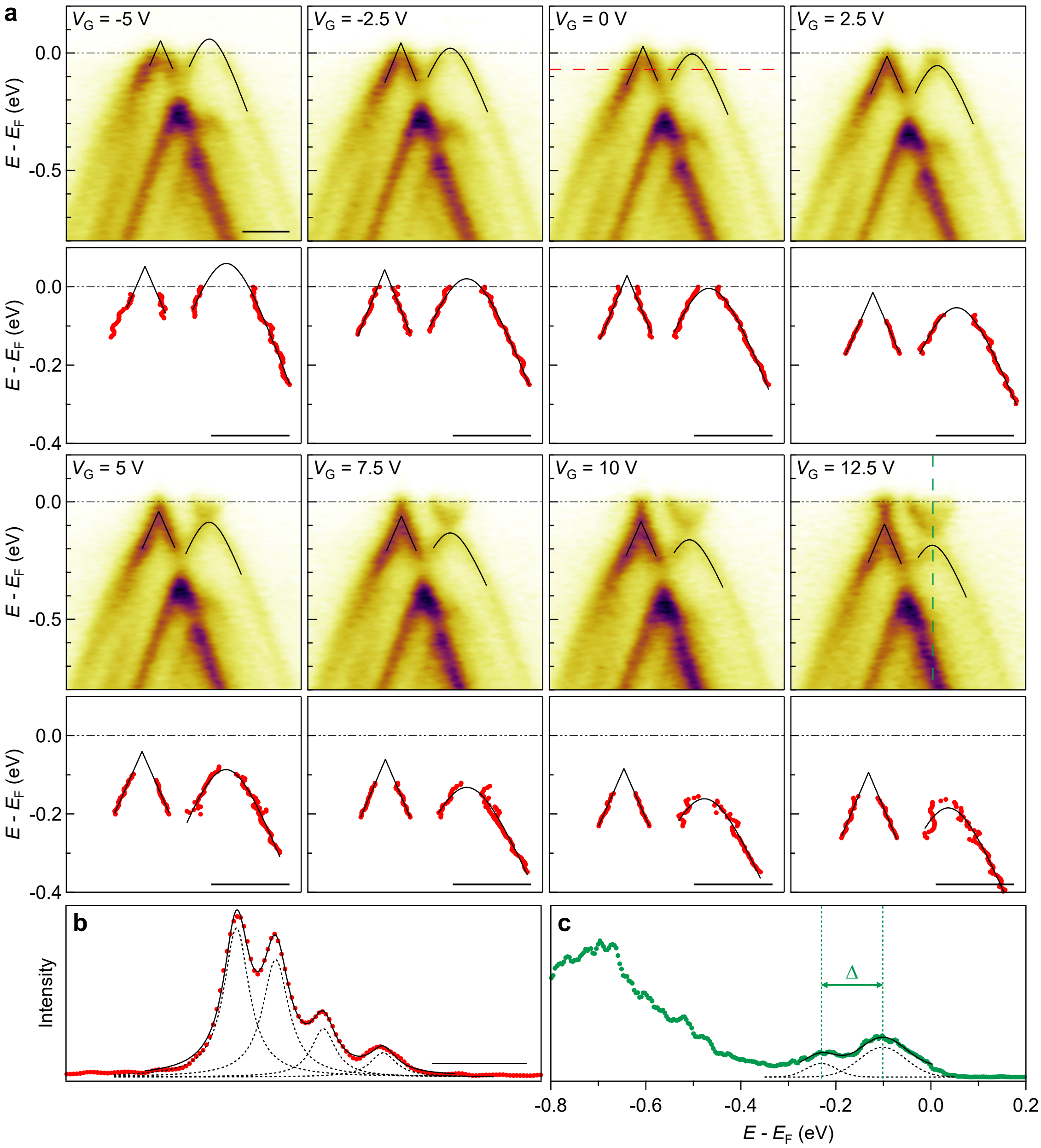

Figure S10: Analysis of tMBG spectra at different gate voltages. **a** ARPES spectra along the κ_1-κ_2 direction for tMBG (top panel) and extracted band positions for the monolayer and bilayer cone (bottom panel) at different gate voltages. Solid lines are fits to the extracted band positions using the low-energy dispersion relations for monolayer and bilayer graphene. **b** MDC extracted along the red horizontal dashed line in the $V_G = 0$ V spectrum. Dashed peaks show individual Lorentzian fits to each band. **c** EDC extracted along the vertical dashed line in the $V_G = 12.5$ V spectrum. Dashed peaks show individual Gaussian fits, the separation between their peak energies provides the bilayer gap size, Δ. All scale bars are 0.1 Å^{-1}.

References

1. Frisenda, R. *et al.* Recent progress in the assembly of nanodevices and van der Waals heterostructures by deterministic placement of 2D materials. *Chem. Soc. Rev.* **47,** 53–68 (2018).
2. Bistritzer, R. & MacDonald, A. H. Moire bands in twisted double-layer graphene. *Proc. Natl. Acad. Sci. USA* **108,** 12233–12237 (2011).
3. Garcia-Ruiz, A., Deng, H.-Y., Enaldiev, V. V. & Fal'ko, V. I. Full Slonczewski-Weiss-McClure parametrization of few-layer twistronic graphene. *Phys. Rev. B* **104,** 085402 (2021).
4. Xu, S. *et al.* Tunable van Hove singularities and correlated states in twisted monolayer–bilayer graphene. *Nat. Phys.* **17,** 619–626 (2021).
5. Yin, J. *et al.* Dimensional reduction, quantum Hall effect and layer parity in graphite films. *Nat. Phys.* **15,** 437–442 (2019).
6. Shirley, E. L., Terminello, L. J., Santoni, A. & Himpsel, F. J. Brillouin-zone-selection effects in graphite photoelectron angular distributions. *Phys. Rev. B* **51,** 13614–13622 (1995).
7. Mucha-Kruczynski, M., Wallbank, J. R. & Fal'ko, V. I. Moiré miniband features in the angle-resolved photoemission spectra of graphene/hBN heterostructures. *Phys. Rev. B* **93,** 085409 (8 2016).
8. Kim, K. *et al.* Van der Waals heterostructures with high accuracy rotational alignment. *Nano Lett.* **16,** 1989–1995 (2016).
9. Lau, C. N., Bockrath, M. W., Mak, K. F. & Zhang, F. Reproducibility in the fabrication and physics of moiré materials. *Nature* **602,** 41–50 (2022).
10. Rosenberger, M. R. *et al.* Nano-"Squeegee" for the Creation of Clean 2D Material Interfaces. *ACS Appl. Mater. Interfaces* **10,** 10379–10387 (2018).
11. McCann, E. & Koshino, M. The electronic properties of bilayer graphene. *Rep. Prog. Phys.* **76,** 056503 (2013).
12. Mucha-Kruczyński, M. *et al.* Characterization of graphene through anisotropy of constant-energy maps in angle-resolved photoemission. *Phys. Rev. B* **77,** 195403 (2008).
13. Zhu, J., Shi, J. & MacDonald, A. H. Theory of angle-resolved photoemission spectroscopy in graphene-based moiré superlattices. *Phys. Rev. B* **103,** 235146 (23 2021).
14. Song, S. M., Park, J. K., Sul, O. J. & Cho, B. J. Determination of Work Function of Graphene under a Metal Electrode and Its Role in Contact Resistance. *Nano Lett.* **12,** 3887–3892 (2012).
15. Damascelli, A. Probing the Electronic Structure of Complex Systems by ARPES. *Phys. Scr.* **T109,** 61–74 (2004).
16. Thompson, J. J. P. *et al.* Determination of interatomic coupling between two-dimensional crystals using angle-resolved photoemission spectroscopy. *Nat. Commun.* **11,** 3582 (2020).
17. Guo, T. *et al.* High-quality-factor flexible and transparent capacitors with Cr–Au nanomeshes as bottom electrodes. *Nanotechnology* **30,** 284001 (2019).
18. Slizovskiy, S. *et al.* Out-of-Plane Dielectric Susceptibility of Graphene in Twistronic and Bernal Bilayers. *Nano Lett.* **21,** 6678–6683 (2021).
19. Laturia, A., Van de Put, M. L. & Vandenberghe, W. G. Dielectric properties of hexagonal boron nitride and transition metal dichalcogenides: from monolayer to bulk. *NPJ 2D Mater. Appl.* **2,** 6 (2018).
20. McCann, E. in *Graphene Nanoelectronics* 237–275 (Springer Berlin Heidelberg, 2011).
